# Ticagrelor Does Not Inhibit Adenosine Transport at Relevant Concentrations: A Randomized Cross-Over Study in Healthy Subjects *In Vivo*

**DOI:** 10.1371/journal.pone.0137560

**Published:** 2015-10-28

**Authors:** T. N. A. van den Berg, S. El Messaoudi, G. A. Rongen, P. H. H. van den Broek, A. Bilos, A. R. T. Donders, M. E. Gomes, N. P. Riksen

**Affiliations:** 1 Department of Pharmacology-Toxicology, Radboud university medical center, Nijmegen, The Netherlands; 2 Department of Internal Medicine (division of vascular medicine), Radboud university medical center, Nijmegen, The Netherlands; 3 Department for Health Evidence, Radboud university medical center, Nijmegen, The Netherlands; 4 Department of Cardiology, Canisius Wilhelmina Hospital, Nijmegen, The Netherlands; Kurume University School of Medicine, JAPAN

## Abstract

**Background and Purpose:**

In patients with myocardial infarction, ticagrelor reduces cardiovascular and sepsis-related mortality, and can cause dyspnea. It is suggested that this is caused by adenosine receptor stimulation, because in preclinical studies, ticagrelor blocks the nucleoside transporter and increases cellular ATP release. We now investigated the effects of ticagrelor on the adenosine system in humans *in vivo*.

**Experimental Approach:**

In a double-blinded, placebo-controlled cross-over trial in 14 healthy subjects, we have tested whether ticagrelor (180 mg) affects adenosine- and dipyridamole-induced forearm vasodilation, as surrogates of nucleoside uptake inhibition and adenosine formation, respectively. Also, *ex vivo* uptake of adenosine and uridine in isolated red blood cells was measured. Primary endpoint was adenosine-induced vasodilation.

**Key Results:**

Ticagrelor did not affect adenosine- or dipyridamole-induced forearm vasodilation. Also, *ex vivo* uptake of adenosine and uridine in isolated red blood cells was not affected by ticagrelor. *In vitro*, ticagrelor dose-dependently inhibited nucleoside uptake, but only at supra-physiological concentrations.

**Conclusion and Implications:**

In conclusion, at relevant plasma concentration, ticagrelor does not affect adenosine transport, nor adenosine formation in healthy subjects. Therefore, it is unlikely that this mechanism is a relevant pleiotropic effect of ticagrelor.

**Trial Registration:**

ClinicalTrials.gov NCT01996735

## Introduction

Ticagrelor is a novel direct-acting and reversibly binding P2Y_12_ receptor antagonist. In the Platelet Inhibition and Patient Outcomes (PLATO) trial, the administration of ticagrelor to patients with an acute coronary syndrome resulted in a striking reduction in the primary endpoint of death from vascular causes, myocardial infarction, or stroke compared to clopidogrel [1]. Moreover, all cause mortality was reduced, and this was driven not only by vascular mortality, but also by fewer deaths attributed to sepsis [2]. In addition, dyspnea and asymptomatic ventricular pauses were reported more often in the ticagrelor treated patients. These finding have instigated the search for pleiotropic effects of ticagrelor over and above the classical antiplatelet effect.

In the past years, evidence is accumulating that ticagrelor inhibits the cellular uptake of the endogenous nucleoside adenosine by blockade of the Equilibrative Nucleoside Transporter (hENT1) [3]. Stimulation of membrane-bound adenosine receptors induces various beneficial cardiovascular effects, including vasodilation, inhibition of platelet aggregation, inhibition of inflammation, and increasing resistance against ischemia and reperfusion [4,5]. In addition, intravenous administration of adenosine induces dyspnea and potently inhibits atrioventricular conductance [5,6]. Adenosine is mainly formed by intra- and extracellular dephosphorylation of AMP, which is catalyzed by 5’-nucleotidase. In contrast, degradation of adenosine in humans is mainly confined to the intracellular compartment, by adenosine kinase and adenosine deaminase. Consequently, in normal situations, the intracellular concentration is lower than the extracellular concentration, and extracellular adenosine is rapidly taken up via the hENT1 by surrounding cells, mainly red blood cells and endothelial cells [7]. Therefore, inhibition of the hENT1 activity will increase the extracellular adenosine concentration, and this mechanism has been proposed to mediate the effects of ticagrelor observed in the PLATO study [3].

Most of the current evidence on the effect of ticagrelor on adenosine metabolism is derived from *in vitro* studies, or from studies in patients in which the adenosine metabolism could be affected by other factors, such as co-medications. Therefore, in this study we aimed to test this hypothesis in healthy humans *in vivo*. We used the vasodilator response to the local administration of adenosine and to forearm ischemia as validated surrogates of adenosine uptake inhibition, as previously described [8–11]. Furthermore, the vasodilator response to dipyridamole, a potent inhibitor of hENT1, was used as a validated surrogate marker of endogenous adenosine formation [12,13]. In addition, the effect of ticagrelor on adenosine and uridine uptake was investigated directly in isolated red blood cells and whole blood.

## Methods

### Subjects

**Fig 1:**
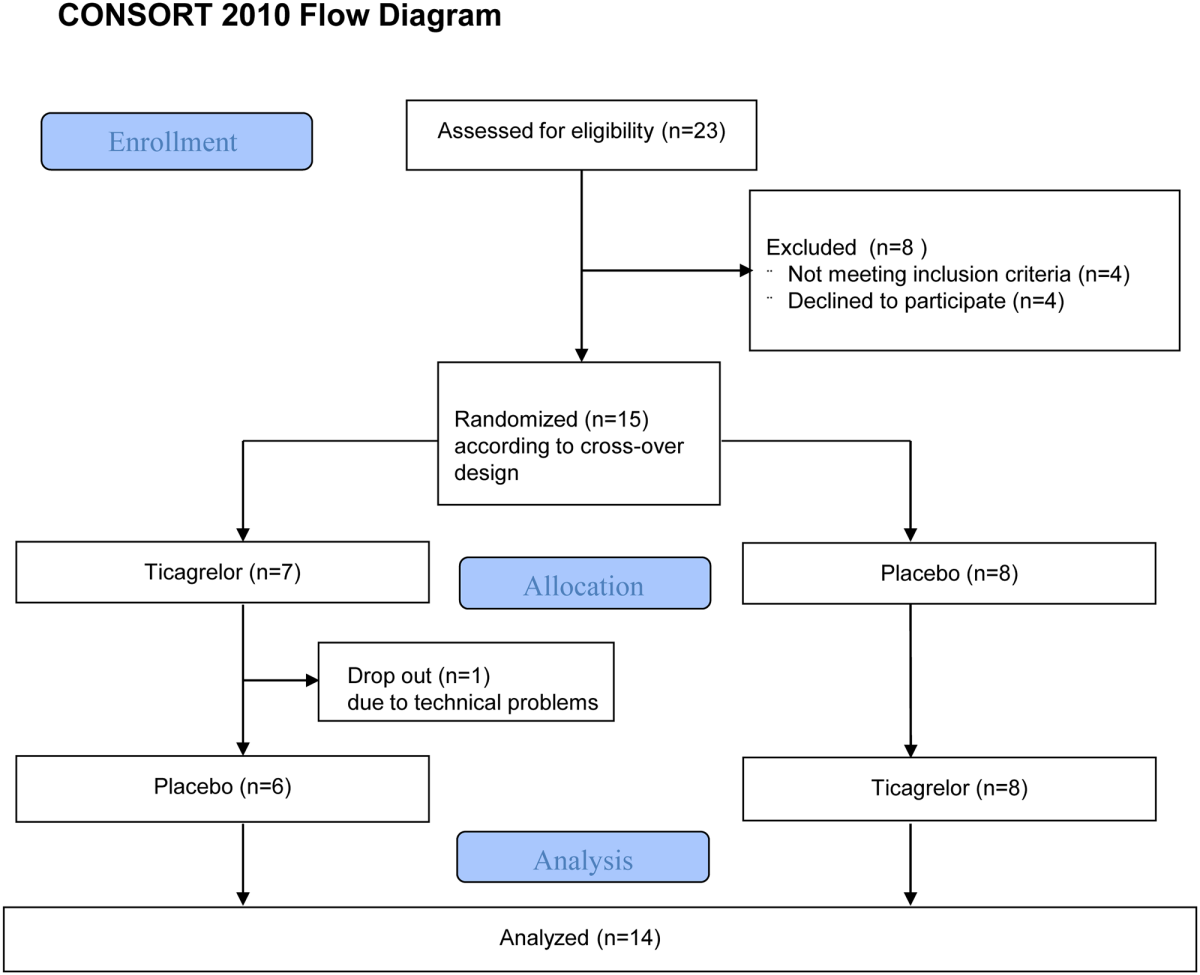
Study Flow chart.

The study protocol was in accordance with the Declaration of Helsinki and was approved by the Institutional Review Board of the Radboud University Medical Center, Nijmegen, The Netherlands. After approval, 15 healthy male volunteers signed written informed consent statements before participation in the study, see also Fig 1. They had no history of cardiovascular disease, bleeding tendency or asthma, and they were all non-smokers. Concomitant medication was not permitted. In all participants a physical examination, electrocardiography, and laboratory investigation were performed to exclude cardiovascular and pulmonary disease, hypertension, diabetes mellitus, renal dysfunction, liver enzyme abnormalities, and thrombocytopenia.

### Experimental protocol

The subjects were randomly assigned in a double-blinded cross-over design to a single dose of ticagrelor 180 mg (Brilique, 2 tabs of 90 mg, AstraZeneca) or 2 fully mimicking placebos (Apotheek Haagse Ziekenhuizen, the Hague, the Netherlands). The experiments were performed two hours after the intake of the study medication. The two experiments were separated by at least 14 days. The randomization code was kept at the department of Clinical Pharmacy of the Radboud University Medical Centre. The study medication was taken under supervision at the Clininal Research Centre of our hospital.

This trial was prospectively registered at: clinicaltrials.gov (The effect of ticagrelor on the adenosine system; NCT01996735).

### Venous occlusion plethysmography

**Fig 2:**
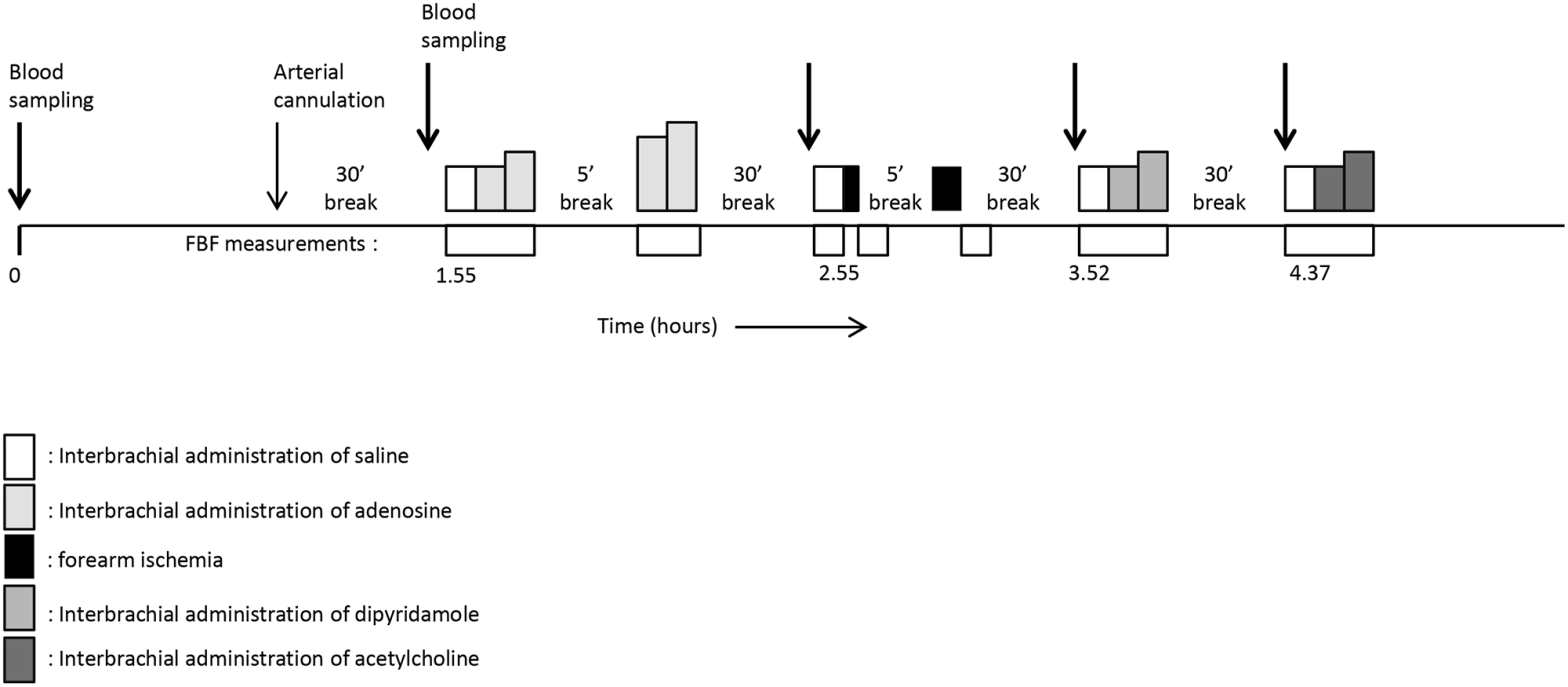
Schematic flow chart of the experiment.

On the experimental days, we studied the subjects in supine position after an overnight fast. All volunteers were asked to abstain from alcoholic drinks and caffeine-containing beverages for at least 24 hours before the experiments. We performed the experiment in a temperature-controlled room (24±1°C). A 27-gauge needle was inserted into the brachial artery of the non-dominant arm for intra-arterial drug administration. We measured the forearm blood flow (FBF) in both forearms simultaneously with venous occlusion plethysmography, using mercury-in-silastic strain gauges and an occluded hand circulation as described previously [14]. The total intra-arterial infused volume was kept at a constant rate of 50 μL/min per dL of forearm volume. Thirty minutes after cannulation of the brachial artery, we started the infusion of normal saline at the calculated rate with concomitant measurement of the FBF. Normal saline (baseline) and the dosages of the intra-arterial administered drug were infused for 5 minutes. We performed four experiments, starting two hours after intake of the study medication, which were all separated by a wash-out period of 30 minutes to prevent any cross-over effects. Fig 2 provides a schematic overview of the experiment.

1. We measured FBF during the administration of 4 incremental dosages of adenosine into the brachial artery. The dosages (0.15, 0.5, 1.5, and 5.0 μg/min/dL forearm volume) are similar to a previous study in which we demonstrated a significant potentiation of adenosine-induced vasodilation by the ENT inhibitor dipyridamole and draflazine [9,10]. Adenosine-induced forearm vasodilation is the primary endpoint of our study. Secondary endpoints include.
2. The blood flow response to 2 and 5 minutes of forearm ischemia was measured (post-occlusive reactive hyperemia; PORH). Forearm ischemia was induced by inflation of an upper arm cuff to 200 mmHg, as described previously [14]. We have previously demonstrated that dipyridamole potentiates the PORH response [8] and that statins potentiate the PORH by an increase of the extracellular adenosine formation [15].
3. Subsequently, FBF was measured during intra-arterial administration of dipyridamole (30 and 100 μl/min/dL forearm volume, which we also used in previous studies [11]). Dipyridamole induces vasodilation by ENT inhibition, thereby increasing the extracellular adenosine concentration at a rate proportionate to extracellular adenosine formation.
4. Finally, we measured the FBF response to the administration of acetylcholine (0.5 and 2.0 μg/min/dL forearm volume) to exclude nonspecific effects on vascular reactivity.

### Blood sampling

Before intake of the study medication blood was drawn from an intravenous cannula in the dominant arm for the determination of the plasma ticagrelor and caffeine concentration, and for the *ex vivo* ENT transport measurements in isolated red blood cells. This measurement was repeated approximately 2 hours after the ingestion of the study medication and just before measurement of the vasodilator response to adenosine. Finally, the ticagrelor concentration was determined before intake of the ticagrelor dose, 2 hours after the intake, and subsequently before the start of each forearm blood flow experiment.

### Analytic procedures

We determined plasma caffeine concentrations using reversed-phase HPLC with ultraviolet detection set at 273 nm, according to Schreiber-Deturmeny and Bruguerolle [16]. In isolated red blood cells, the uptake of adenosine and uridine was determined as described previously [17]. In contrast to adenosine, uridine is not metabolized inside the cells, and therefore, uridine uptake is a more direct measure of ENT activity than adenosine uptake [18]. Ticagrelor concentrations were determined by LC-MSMS. Liquid chromatographic separation was performed at a temperature of 30°C with a mobile phase, consisting of solvent A (10 mM ammonium acetate in water) and solvent B (acetonitrile). For the mass spectrometric analysis, heated electrospray ionization (HESI) was operated at a spray voltage of -2.5kV, a capillary temperature of 225°C and a vaporizer temperature of 382°C. Argon was used as collision gas at a pressure of 1.5 mTorr. Negative ion mode was used with selected reaction monitoring (SRM) for the quantitative analysis of ticagrelor. The most abundant product ion was used for quantification. The quantification was performed using peak areas.

### In vitro experiments

In isolated red blood cells from healthy volunteers not taking any medication, the effect of ticagrelor on uridine uptake was measured as described previously (17). Briefly, uridine was added to washed red blood cells to obtain a final concentration of 1000 μM. The cells were pre-incubated with increasing concentrations of ticagrelor for ten minutes before the addition of uridine. After 3 seconds, uridine uptake was completely blocked by the addition of 10 μM dipyridamole and the amount of uridine in the cell was determined using HPLC with UV detection set at 254 nm.

Because of the high protein binding of ticagrelor of 99.8%, we also performed whole blood experiments, in which ticagrelor was added to 1 ml of whole blood for 10 or 60 minutes. Next, the red blood cells were isolated by centrifugation and uridine was added as described above.

To measure the rate of disappearance of adenosine in the physiological situation, which is the overall result of uptake and intracellular degradation, we added 1 μM of adenosine to 1 ml of whole blood at 37°C, as previously described by Bonello et al [19]. After 0, 15, 30, 45, and 60 seconds, the transport, formation, and degradation of adenosine was completely blocked by a adding an equal volume of pharmacological blocking solution, including NaCl (118mM); KCl (5 mM); Na_2_EDTA (13.2 mM); dipyridamole (40 μM); iodotubercidin (ITU; 10μM); EHNA (10μM); forskolin (11.5μM), and IBMX (115μM). After centrifugation the adenosine concentration was measured in the supernatant using LC-MSMS. Separation was performed with a Acquity UPLC HSS column. The mobile phase, consisting of solvent A (1 mM ammonium fluoride in water) and solvent B (methanol). For the mass spectrometric analysis, heated electrospray ionization (HESI) was operated at a spray voltage of +3kV, the capillary temperature and the vaporizer temperature were set at 300°C. Argon was used as collision gas at a pressure of 1.5 mTorr. Positive ion mode was used with selected reaction monitoring (SRM) for the quantitation. The following SRM transitions were used: *m/z* 268.1(parent ion) to *m/z* 119.0 and 136.1 (both product ions).

### Statistical analysis

The study was powered to detect a difference in the primary endpoint of adenosine-induced vasodilation. The sample size calculation was based on the following assumptions: in previous studies from our own group, we found that the vasodilator response to the intrabrachial administration of adenosine averages 2.8±0.6, 4.4±1.0, 9.0±1.7 ml/min per dl of forearm volume for 0.5, 1.5, and 5.0 μg/min/dl, respectively (mean±SEM, n = 8). The pooled CV is 0.6, so after log transformation the SD averaged 0.55. We expect that a (relative) difference between the treatments is independent of the adenosine dose. Hence, a linear mixed model will be used, with fixed factors treatment (ticagrelor vs placebo), adenosine dose, and period. Based on a correlation of 0.7 between the measurements for all doses and time points and an SD of 0.55, the SD of the contrast is 0.25. As a result, 13 evaluable subjects are needed to demonstrate an augmentation of adenosine-induced vasodilation with 1.25 (ie a 25% increase) with a power of 80% and a two-sided alpha of 0.05. To account for one drop-out, we aim to include 14 healthy volunteers.

FBF analyses were done offline before unblinding of the study. Mean FBF values were calculated from the FBF responses to the different stimuli. We calculated the average FBF during last 4 minutes of the baseline FBF (normal saline), the last 2 minutes of the FBF response to adenosine, dipyridamole and acetylcholine, and the first 3 minutes of the PORH. Results are expressed as the median absolute FBF (mL/dL/min) with interquartile range (25–75%), unless otherwise stated.

A linear mixed model was used to compare the FBF results of both experiments, with the log FBF during placebo and ticagrelor treatment as the dependent variable, and treatment (ticagrelor versus placebo), adenosine dose, and period as fixed factors. We used a heterogeneous compound symmetry as the type of repeated covariance. A carry over effect was excluded calculating the interaction between ‘sequence’ and the primary endpoint in the mixed model analysis. For uridine uptake experiments, sigmoidal dose response curves were constructed with a variable slope and IC_50_ were calculated for each series of experiments, using GraphPad Prism.

## Results

### Subjects

23 subjects were screened for eligibility. Four participants withdrew from participation and 4 participants were excluded, because of drug abuse, a history of asthma, a platelet count of 145*10^9^/L, and a systolic heart murmur. Insertion of the arterial needle failed in 1 subject and this subject was replaced. Due to repeated dislocation of the intra-arterial needle, we had to discontinue the experiment after the infusion of dipyridamole during a single visit in another subject. Thus, 13 fully evaluable subjects were included and one subject in whom acetylcholine responses were lacking during one (placebo) visit, see also Fig 1.

The baseline characteristics are depicted in Table 1. The plasma caffeine concentration was < 1 mg/L in all subjects, showing adequate caffeine restriction. The ticagrelor concentration averaged 1.18±0.13, 1.01±0.11, 0.90±0.09, and 0.80±0.08 μM (mean±SE) at 2, 3, 4, and 4.5 hours after ticagrelor intake, respectively (see Fig 2 for the time points).

**Table 1:** Baseline characteristics (means ± SD; n = 14).

Characteristic	Value
Age (years)	22.1 ± 2.2
Blood pressure (mmHg)	
Systolic	125 ± 7
Diastolic	67 ± 10
Heart rate (bpm)	62 ± 10
BMI (kg/m^2^)	22.3 ± 1.9
Blood plasma values	
Platelet count (*10^9^/L)	230 ± 31
Creatinine (μmol/L)	80 ± 7
Non-fasting glucose (mmol/L)	5.1 ± 0.7
ALAT (U/L)	29 ± 14
Non-fasting cholesterol (mmol/L)	3.9 ± 0.7

### Venous occlusion plethysmography

**Fig 3:**
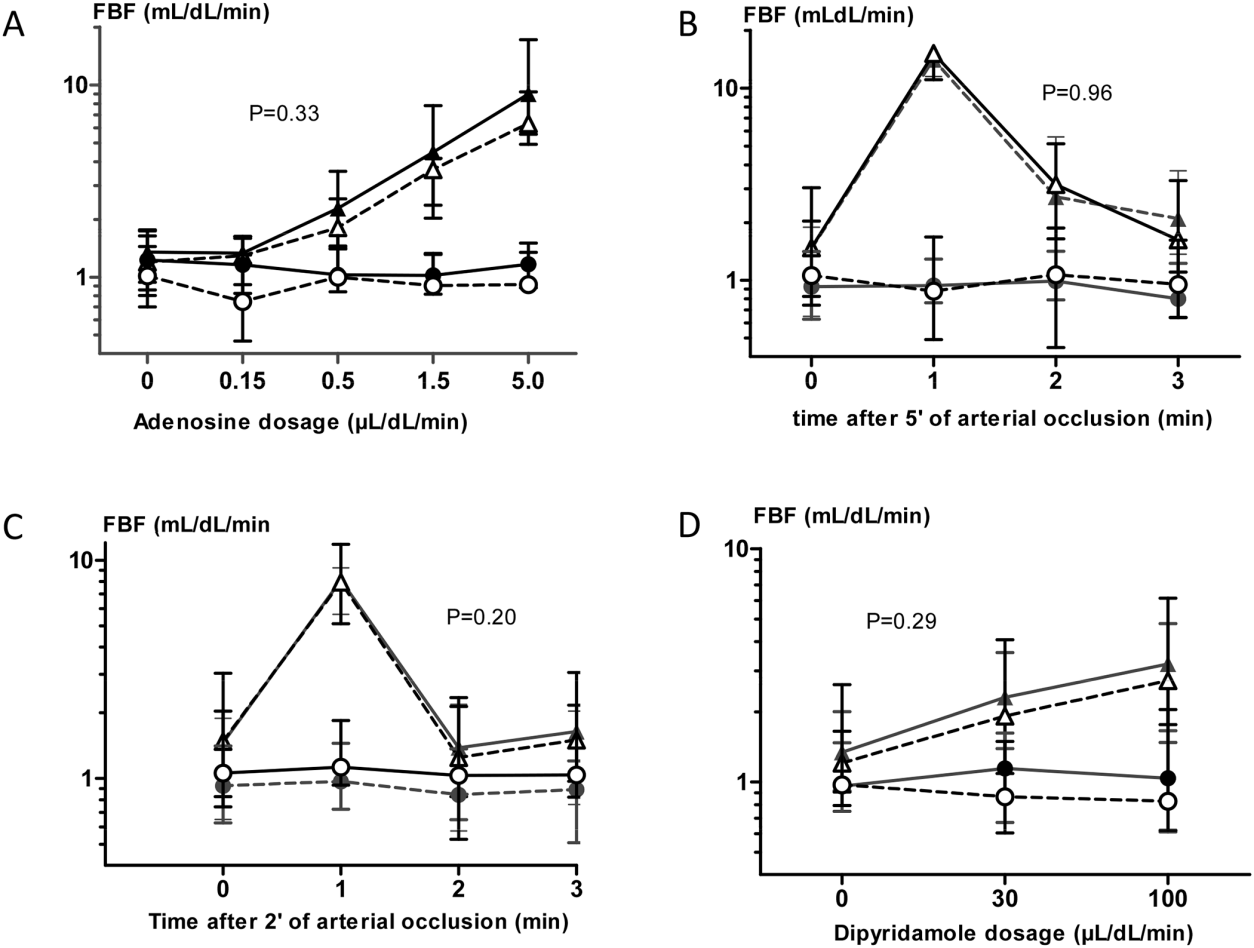
Forearm blood flow measurements. FBF values in the experimental forearm (triangle) and non-experimental forearm (circle) after ticagrelor treatment (black symbols) or placebo treatment (open symbols, dashed line) during intrabrachial administration of adenosine (A), after five (B) and two (C) minutes of forearm ischemia, and during intrabrachial administration of dipyridamole (D). P-values represent the effect of ticagrelor on the FBF-values.

**Fig 4:**
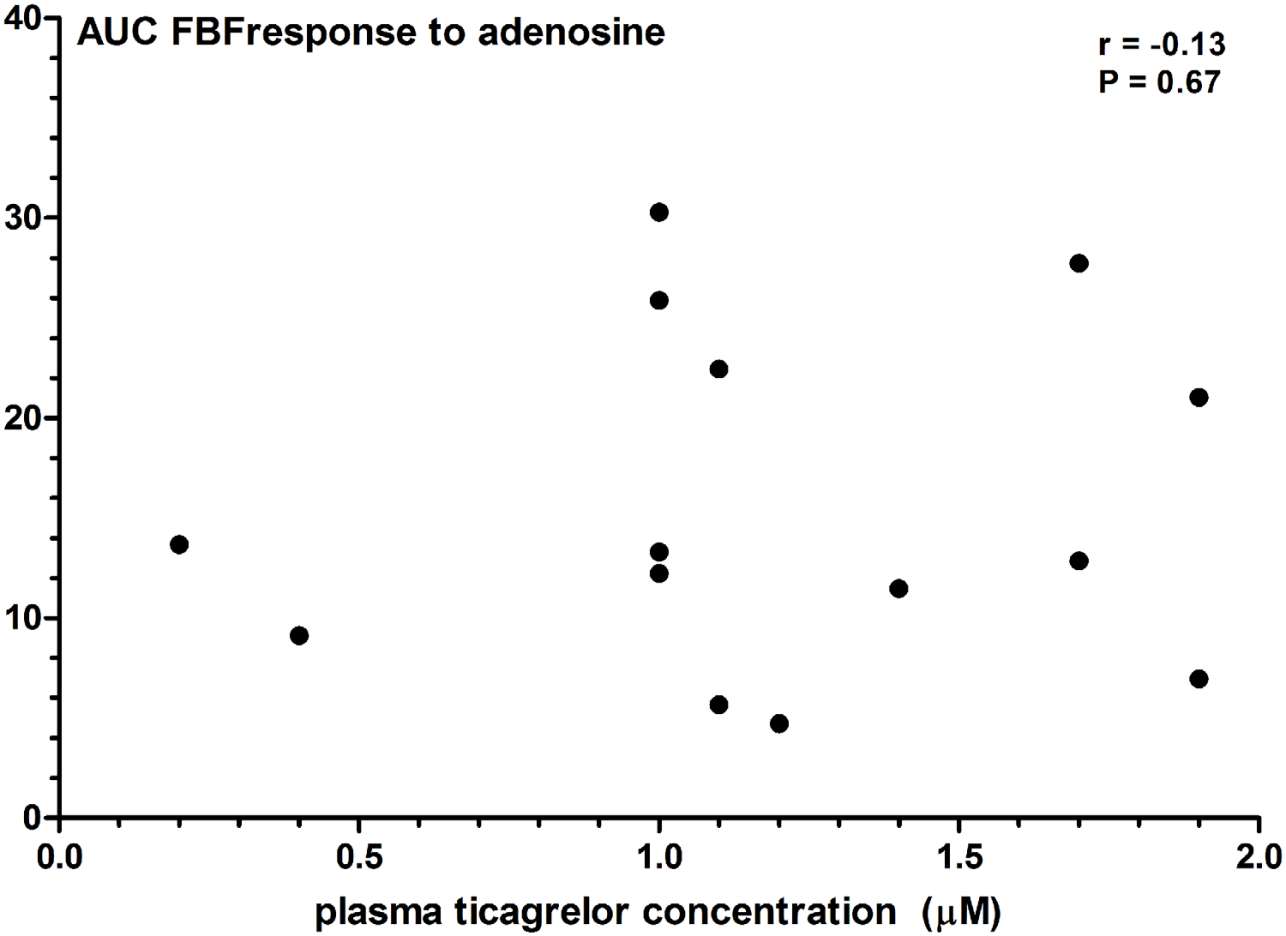
The correlation between the plasma ticagrelor concentration and the area under the curve (AUC) of adenosine-induced forearm vasodilation.

Baseline FBF in the experimental forearm was 1.2 (0.9–1.8) and 1.35 (0.9–1.7) mL/dL/min during placebo and ticagrelor, respectively. There was no significant carry-over effect in this study (P = 0.58). Intrabrachial administration of adenosine significantly increased FBF in the experimental forearm, but not in the control forearm (P<0.01; Fig 3A). Pretreatment with ticagrelor did not potentiate adenosine-induced vasodilation: the incremental dosages of adenosine increased the FBF to 1.3 (0.9–1.6), 1.8 (1.4–2.6), 3.6 (2.0–4.1), and 6.3 (4.9–9.2) mL/dL/min during placebo, and to 1.3 (1.2–1.6), 2.3 (1.5–3.6), 4.5 (2.4–7.8), and 9.0 (5.5–17.2) mL/dL/min after ticagrelor administration (P = 0.33; Fig 3A). Furthermore, we did not see any correlation between the plasma ticagrelor concentrations and the area under the curve for adenosine-induced FBF (Spearman correlation coefficient -0.13, P = 0.67; Fig 4).

Baseline FBF before arterial occlusion was 1.5 (0.8–3.0) mL/dL/min during placebo and 1.5 (0.6–1.9) ml/dl/min during ticagrelor treatment. The hyperemic response after 2 minutes of arterial occlusion was 7.9 (5.1–11.8), 1.2 (0.8–2.3), and 1.5 (1.0–3.1) during placebo and 8.1 (5.6–9.2), 1.4 (0.6–2.2), and 1.6 (0.8–2.0) during ticagrelor in the 1^st^, 2^nd^ and 3^rd^ minute respectively (*p* = 0.96 for the effect of ticagrelor) (Fig 3B).

After 5 minutes of arterial occlusion, the hyperemic response was 15.2 (11.1–23.7), 3.1 (1.6–5.1), and 1.6 (1.1–3.3) mL/dL/min during placebo and 14.1 (11.5–20.3), 2.7 (1.9–5.6), and 2.1 (1.4–3.7) mL/dL/min in the 1^st^, 2^nd^ and 3^rd^ minute respectively (*p* = 0.20 for effect of ticagrelor; Fig 3C).

Comparable to adenosine, intrabrachial administration of dipyridamole significantly increased FBF in the experimental forearm but not in the control forearm (P<0.01, Fig 3D). Again, the vasodilator response to dipyridamole did not differ between placebo and ticagrelor treatment. The FBF in the experimental forearm was 1.2 mL/dL/min (0.9–2.6) at baseline and 1.9 (1.1–4.1) and 2.7 (1.8–6.1) during administration of the two doses of dipyridamole after treatment with placebo, versus 1.3 (0.9–2.0), 2.3 (1.4–3.6), and 3.2(1.5–4.8) after treatment with ticagrelor (*p* = 0.29 for effect of ticagrelor, Fig 3D).

Similarly, ACh-induced vasodilation did not differ between both experiments. The FBF at baseline and during administration of the two doses of acetylcholine was 3.5 mL/dL/min (2.0–5.3), 10.8 (7.8–19.1), and 17.3 (12.5–31.2) after treatment with placebo, versus 3.9 (3.0–6.5), 8.1 (4.4–19.3), and 16.8 (10.4–28.6) after treatment with ticagrelor (*p* = 0.11 for effect of ticagrelor).

### Ex vivo nucleoside uptake inhibition

**Fig 5:**
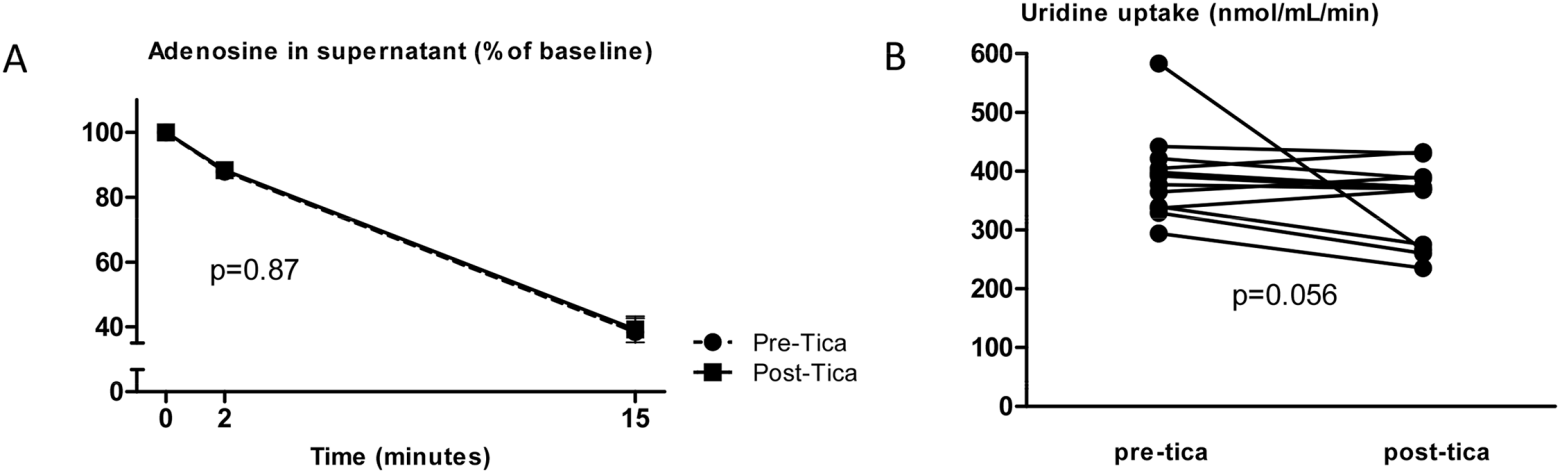
*Ex vivo* uptake of adenosine (A) and uridine (B) in red blood cells isolated from the subjects before (pre-tica) and two hours after intake of ticagrelor (post-tica). In A, the adenosine concentration in the supernatant is expressed as the percentage of the baseline adenosine concentration (n = 13; P >0.1). In B, the uridine uptake is expressed for each individual subjects before and after intake of ticagrelor (n = 13, P = 0.056 for comparison between before and after).

Red blood cells were isolated from blood samples taken before and 2 hours after intake of the study drug. The uptake of adenosine in these cells was not affected by oral ticagrelor, when compared to baseline (P = 0.87; Fig 5A). Uridine uptake was inhibited by an average of 11%, but this did not reach statistical significance (P = 0.056) (Fig 5B).

### In vitro nucleoside uptake inhibition

**Fig 6:**
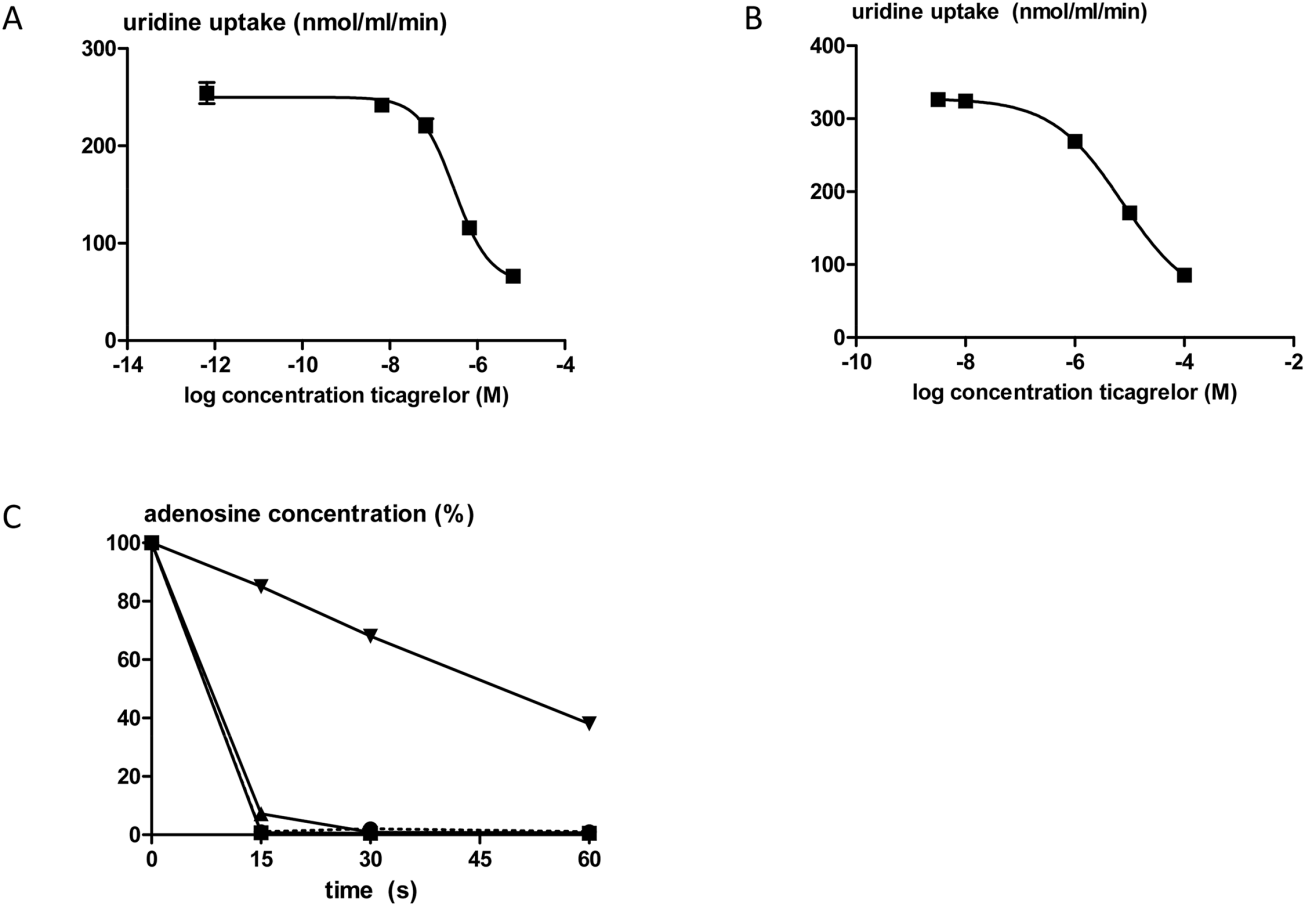
*In vitro* uptake of uridine in isolated red blood cells from healthy subjects. The cells were pre-incubated with ticagrelor either after isolation (A; n = 3) of in whole blood before isolation of the red blood cells (B; n = 3). In C, the adenosine disappearance in whole blood from healthy volunteers is expressed as the percentage of the baseline concentration in the presence of ticagrelor in the concentrations 10^−6^ M (squares), 10^−5^ M (triangles), and 10^−4^ M (reversed triangles or in the presence of vehicle (circles, dashed line) (n = 2).

In isolated red blood cells, ticagrelor dose-dependently inhibited uridine uptake with an IC_50_ value of 3.0*10^−7^ M (95% CI 2.0*10^−7^ to 4.5*10^−7^, n = 3; Fig 6A). In the experiments in which ticagrelor was added to whole blood before isolation of the red blood cells, the IC_50_ of ticagrelor for uridine uptake inhibition was 7.3*10^−6^ M (95% CI 6.0*10^−6^ to 1.3*10^−5^, n = 3; Fig 6B). In addition, in whole blood, the disappearance of adenosine was only inhibited significantly by the highest concentration of ticagrelor used (10^−4^ M) (n = 2; Fig 6C).

## Discussion & Conclusions

The main finding of our study is that in healthy subjects a single dose of 180 mg of ticagrelor does not potentiate the forearm vasodilator response to adenosine, nor post-occlusive reactive hyperemia and dipyridamole-induced vasodilation. In addition, we observed no significant *ex vivo* nucleoside uptake inhibition in isolated red blood cells in these subjects after oral treatment with ticagrelor. These *in vivo* and *ex vivo* findings are consistent with additional *in vitro* studies in isolated red blood cells demonstrating that ticagrelor only inhibits nucleoside transport in concentrations that are considerably higher than those obtained after normal dosing. Together, our findings argue against a role for increased adenosine receptor stimulation as a relevant pleiotropic effect of ticagrelor.

In the last few years, several papers suggest that ticagrelor increases the extracellular concentration of endogenous adenosine and it is speculated that this contributes to the effects of ticagrelor that are observed in clinical studies that cannot easily be explained by platelet aggregation inhibition, including reduced mortality, dyspnea, ventricular pauses, limitation of myocardial infarct size, and effects on inflammation [3,20]. Adenosine induces various effects by stimulation of membrane-bound adenosine receptors, including direct vasodilation, platelet aggregation inhibition, modulation of sympathetic nervous system activity, modulation of inflammation, and limitation of (myocardial) ischemia and reperfusion injury [4,5]. In addition, the intravenous administration of adenosine has negative dromotropic effects on the heart, and induces a sensation of dyspnea [5,6].

Previous *in vitro* studies have demonstrated two different effects of ticagrelor on adenosine metabolism: first, ticagrelor augments the release of adenosine triphosphate (ATP), which acts as a precursor for extracellular adenosine formation, from isolated red blood cells, with an IC_50_ value of 14 μM [21]. Secondly, ticagrelor inhibits adenosine uptake via the ENT with a reported IC_50_ value of 100–260 nmol/l [22,23]. It needs to be emphasized, however, that these experiments were performed in isolated cells without plasma. Given the > 99% plasma protein binding of ticagrelor in human blood, these IC_50_ values cannot easily be compared to the whole blood ticagrelor concentration of 0.5–1.5 μM observed during normal dosing in patients. Ticagrelor does not have a direct effect on adenosine receptors itself, does not affect the breakdown of adenosine by adenosine deaminase, nor is it converted into adenosine [19].

Our experiment was designed to investigate both the effect of ticagrelor on adenosine uptake as well as the effect of ticagrelor on extracellular adenosine formation (e.g. by increased ATP release). Direct measurement of the extracellular adenosine concentration is technically highly demanding given the extremely short half-life of adenosine and there is great controversy about the normal extracellular concentration in human plasma [24]. Therefore, we used well-validated surrogates of extracellular adenosine formation and uptake. In the absence of any effects of ticagrelor on adenosine receptors or adenosine degradation, the vasodilator response to the intrabrachial administration of adenosine is a well-validated surrogate of adenosine transporter function, as we have previously described [9,10]. We also studied PORH because this is mediated, at least in part, by an increased endogenous formation of adenosine [8]. Dipyridamole is a potent hENT1 blocker and increases the extracellular adenosine concentration at a rate proportional to the extracellular formation of adenosine, as demonstrated previously [12]. We did not observe an effect of ticagrelor on any of these stimuli, demonstrating that there is no relevant increase in extracellular adenosine formation and adenosine transport. Nonspecific effects of ticagrelor on vascular reactivity were ruled out using acetylcholine-induced vasodilation. Consistent with these *in vivo* findings, there was no significant inhibition of *ex vivo* adenosine uptake in red blood cells, isolated before and after ticagrelor-intake. Uridine uptake was inhibited with 11%, but this did not reach statistical significance (P = 0.056). For the interpretation of these results, it is important to realize that after facilitated diffusion via the ENT, adenosine is rapidly metabolized, and that intracellular deamination of adenosine is rate limiting for adenosine uptake rather than ENT activity [25]. Only after pharmacological inhibition of >80% of ENT activity, the ENT transporter becomes rate limiting, and adenosine uptake is reduced [25]. As such, 11% ENT inhibition will not have any effect on extracellular adenosine concentration. Indeed, also in whole blood experiments, ticagrelor did not affect adenosine disappearance at relevant concentrations.

The results of our series of experiments contradict previous studies. *In vitro* Van Giezen et al reported in a canine model that ticagrelor augmented adenosine-induced vasodilation in the coronary artery [22]. However, this effect was observed only at a ticagrelor plasma concentration of 13.4 μM and not with the lower concentration of 4.1 μM, which is still considerably higher than the plasma concentration in patients treated with ticagrelor. In contrast, in another in vitro study, the addition of 1 μM of ticagrelor to whole blood resulted in a slightly but significantly higher adenosine concentration 1 minute after the addition of 7.1 μM of adenosine [26]. In healthy subjects *in vivo* Wittfeldt et al showed that a single dose of 180 mg of ticagrelor augmented the coronary blood flow velocity after intravenous adenosine infusion, which was prevented by concomitant administration of the adenosine receptor antagonist theophylline [27]. Similar results were obtained by Alexopoulos et al in patients with a recent non-ST-elevation acute coronary syndrome [28]. The route of administration in these studies is the major and important difference with our study: in our model adenosine is administered into the brachial artery, resulting in a high local arterial adenosine concentration, but without any systemic effects. Intravenous administration of adenosine, however, induces major systemic hemodynamic effects consisting of increased systolic blood pressure and heart rate and decrease of diastolic blood pressure by the combination of a direct vasodilator effect and a pronounced activation of sympathetic nervous system activity by stimulation of peripheral chemoreceptors [29]. Therefore, the coronary blood flow response in the Wittfeldt and Alexopoulos papers is driven by different effects, whereas adenosine-induced vasodilation in our study was not confounded by systemic hemodynamic and nervous effects. Finally, the plasma concentration of ticagrelor in the study by Wittfeldt et al was slightly higher than in our study, despite the fact that in both studies the plasma concentration was determined 2 hours after intake of 180 mg ticagrelor in healthy subjects. Bonello et al measured plasma adenosine concentration in patients with an acute coronary syndrome who were randomized to either ticagrelor or clopidogrel at a time point of 6 hours after the loading dose [19]. They observed a significantly higher adenosine concentration in the patients treated with ticagrelor, and they observed a significant inhibition of *ex vivo* adenosine uptake in whole blood. It is likely that in the patients in this study, just as in the study by Alexopoulos et al, the adenosine metabolism is affected by the myocardial ischemia, the profound activation of the sympathetic nervous system by the coronary event, and by the comedication (e.g. statins that will increase the extracellular formation of adenosine by activation of the ecto-5’-nucleotidase [12,15]). In addition, the pharmacological blocking solution that is used to completely block adenosine metabolism in the *ex vivo* whole blood experiments differs from the solution that we previously have validated to result in a almost complete recovery of adenosine [24]. The inconsistent results of our study and the Bonello study might be caused, at least in part, by these differences.

### Study limitations

Our study has several potential limitations. First, our experiments were performed in healthy subjects without cardiovascular disease and without concomitant cardiovascular drugs, such as in the study by Bonello et al [19]. Therefore, the results of our study do not exclude any effect of ticagrelor on the adenosine system in patients with acute coronary events. Secondly, we investigated adenosine formation and uptake in the forearm, in contrast to the coronary circulation that was studied by Wittfeldt et al [27]. However, ENT is not known to differ between heart and forearm and therefore we believe that extrapolation from forearm to heart is valid for this particular research question.

### Conclusions

Ticagrelor dose-dependently inhibits nucleoside transporter activity in isolated red blood cells of healthy volunteers. However, the plasma concentration of ticagrelor after normal dosing in humans is too low to result in a significant increase in extracellular adenosine and adenosine receptor stimulation via this mechanism. In addition, the lack of any effect of ticagrelor on dipyridamole-induced vasodilation suggests that ticagrelor does not augment endogenous extracellular adenosine formation. Our findings, therefore, argue against a role these mechanisms as an explanation for the effects of ticagrelor, including lower mortality, dyspnea, ventricular pauses, and modulation of inflammation, as observed in recent clinical studies.

## Supporting Information

S1 Table CONSORT 2010 checklist of information to include when reporting a randomised trial. (DOCX)

S1 Text Trial study protocol: The effect of ticagrelor on the adenosine system. Final version of the study protocol approved by the Institutional Review Board.
(PDF)

## Abbreviations

Ach: acetylcholine
AMP: adenosine monophosphate
ATP: adenosine triphosphate
ENT: Equilibrative Nucleoside Transporter
FBF: forearm blood flow
HPLC: high-performance liquid chromatography
LC-MSMS: liquid chromatography tandem mass spectrometry
PORH: post-occlusive reactive hyperemia
