# Supplementary material for: Ticagrelor Does Not Inhibit Adenosine Transport at Relevant Concentrations: A Randomized Cross-Over Study in Healthy Subjects In Vivo
Source: PLoS One. 2015 Oct 28;10(10):e0137560. doi: 10.1371/journal.pone.0137560 (PMC4624811; doi:10.1371/journal.pone.0137560)
Supplement: S1 Text — Final version of the study protocol approved by the Institutional Review Board. (PDF) [file pone.0137560.s002.pdf]

The effect of ticagrelor on the adenosine system

(July 2012)

PROTOCOL TITLE 'The effect of ticagrelor on the adenosine system'

**Protocol ID**	NL43379.091.13
**Short title**	Ticagrelor and adenosine
**EudraCT number**	2012-004560-21
**Version**	2013 February 28th
**Date**	21-01-2013
**Principal investigator(s) (in Dutch: hoofdonderzoeker/ uitvoerder)**	Dr. Niels P. Riksen<br>Prof. dr. Gerard Rongen<br>Drs. Saloua El Messaoudi<br>Dept. Of Pharmacology-Toxicology 149<br>Radboud University Nijmegen Medical Centre<br><br>Dr. Marc Gomes<br>Dept. of Cardiology<br>Canisius Wilhelmina Hospital Nijmegen
**Sponsor (in Dutch: verrichter/opdrachtgever)**	Radboud University Nijmegen Medical Centre
**Subsidising party**	AstraZeneca
**Independent expert (s)**	Dr. Bas Bredie, internist<br>Dept. of Internal Medicine 463<br>Radboud University Nijmegen Medical Centre
**Laboratory sites**	Dept. of Pharmacology-Toxicology 149

	Radboud University Nijmegen Medical Centre
**Pharmacy**	Dept. of Clinical Pharmacy<br><br>Radboud University Nijmegen Medical Centre

PROTOCOL SIGNATURE SHEET

Name	Signature	Date
**Head of Department:**<br>Prof. dr. Frans Russel<br>Head of department of Pharmacology-Toxicology<br>Radboud University Nijmegen Medical Centre		
**Principal Investigators**<br>Dr. Niels Riksen<br><br>Prof. dr. Gerard Rongen<br><br>Dr. Marc Gomes<br><br>Drs. Saloua El Messaoudi		

TABLE OF CONTENTS

1. INTRODUCTION AND RATIONALE 98
2. OBJECTIVES 119
3. STUDY DESIGN 1110
4. STUDY POPULATION 1511
   4.1 Population (base) 1511
   4.2 Inclusion criteria 1511
   4.3 Exclusion criteria 1511
   4.4 Sample size calculation 1611
5. TREATMENT OF SUBJECTS 1712
   5.1 Investigational product/treatment 1712
   5.2 Use of co-intervention (if applicable) 1712
   5.3 Escape medication (if applicable) 1712
6. INVESTIGATIONAL PRODUCT 1813
   6.1 Name and description of investigational product(s) 1813
   6.2 Summary of findings from non-clinical studies 1813
   6.3 Summary of findings from clinical studies 1813
   6.4 Summary of known and potential risks and benefits 1913
   6.5 Description and justification of route of administration and dosage 2013
   6.6 Dosages, dosage modifications and method of administration 2013
   6.7 Preparation and labelling of Investigational Medicinal Product 2013
   6.8 Drug accountability 2013
7. NON-INVESTIGATIONAL PRODUCT 2214
   7.1 Name and description of non-investigational product(s) 2214
   7.2 Summary of findings from non-clinical studies 2214
   7.3 Summary of findings from clinical studies 2214
   7.4 Summary of known and potential risks and benefits 2214
   7.5 Description and justification of route of administration and dosage 2214
   7.6 Dosages, dosage modifications and method of administration 2314
   7.7 Preparation and labelling of Non Investigational Medicinal Product 2314
   7.8 Drug accountability 2314
8. METHODS 2415
   8.1 Study parameters/endpoints 2415
      8.1.1 Main study parameter/endpoint 2415
      8.1.2 Secondary study parameters/endpoints (if applicable) 2415
      8.1.3 Other study parameters (if applicable) 2415
   8.2 Randomisation, blinding and treatment allocation 2415
   8.3 Study procedures 2415
   8.4 Withdrawal of individual subjects 2515
      8.4.1 Specific criteria for withdrawal (if applicable) 2515
   8.5 Replacement of individual subjects after withdrawal 2515
   8.6 Follow-up of subjects withdrawn from treatment 2515

8.7 Premature termination of the study 2515
9. SAFETY REPORTING 2616
9.1 Section 10 WMO event 2616
9.2 AEs, SAEs and SUSARs 2616
9.2.1 Adverse events (AEs) 2616
9.2.2 Serious adverse events (SAEs) 2616
9.2.3 Suspected unexpected serious adverse reactions (SUSARs) 2717
9.3 Annual safety report 2818
9.4 Follow-up of adverse events 2819
9.5 [Data Safety Monitoring Board (DSMB) / Safety Committee] 2819
10. STATISTICAL ANALYSIS 2920
10.1 Primary study parameter(s) 2920
10.2 Secondary study parameter(s) 2920
10.3 Other study parameters 2920
10.4 Interim analysis (if applicable) 2920
11. ETHICAL CONSIDERATIONS 3021
11.1 Regulation statement 3021
11.2 Recruitment and consent 3021
11.3 Objection by minors or incapacitated subjects (if applicable) 3021
11.4 Benefits and risks assessment, group relatedness 3021
11.5 Compensation for injury 3021
11.6 Incentives (if applicable) 3122
12. ADMINISTRATIVE ASPECTS, MONITORING AND PUBLICATION 3223
12.1 Handling and storage of data and documents 3223
12.2 Monitoring and Quality Assurance 3223
12.3 Amendments 3223
12.4 Annual progress report 3324
12.5 End of study report 3324
12.6 Public disclosure and publication policy 3325
13. STRUCTURED RISK ANALYSIS 3426
13.1 Potential issues of concern 3426
13.2 Synthesis 3527
14. REFERENCES 3527

LIST OF ABBREVIATIONS AND RELEVANT DEFINITIONS

**ABR**	**ABR form, General Assessment and Registration form, is the application form that is required for submission to the accredited Ethics Committee (In Dutch, ABR = Algemene Beoordeling en Registratie)**
**AE**	**Adverse Event**
**AR**	**Adverse Reaction**
**CA**	**Competent Authority**
**CCMO**	**Central Committee on Research Involving Human Subjects; in Dutch: Centrale Commissie Mensgebonden Onderzoek**
**CV**	**Curriculum Vitae**
**DSMB**	**Data Safety Monitoring Board**
**EU**	**European Union**
**EudraCT**	**European drug regulatory affairs Clinical Trials**
**GCP**	**Good Clinical Practice**
**IB**	**Investigator's Brochure**
**IC**	**Informed Consent**
**IMP**	**Investigational Medicinal Product**
**IMPD**	**Investigational Medicinal Product Dossier**
**METC**	**Medical research ethics committee (MREC); in Dutch: medisch ethische toetsing commissie (METC)**
**(S)AE**	**(Serious) Adverse Event**
**SPC**	**Summary of Product Characteristics (in Dutch: officiële productinfomatie IB1-tekst)**
**Sponsor**	**The sponsor is the party that commissions the organisation or performance of the research, for example a pharmaceutical company, academic hospital, scientific organisation or investigator. A party that provides funding for a study but does not commission it is not regarded as the sponsor, but referred to as a subsidising party.**
**SUSAR**	**Suspected Unexpected Serious Adverse Reaction**
**Wbp**	**Personal Data Protection Act (in Dutch: Wet Bescherming Persoonsgevens)**
**WMO**	**Medical Research Involving Human Subjects Act (in Dutch: Wet Medisch-wetenschappelijk Onderzoek met Mensen**

SUMMARY

Rationale: Preclinical studies have shown that the P2Y12 receptor antagonist ticagrelor can increase the extracellular concentration of the endogenous nucleoside adenosine by inhibiting the cellular uptake of adenosine via the equilibrative nucleoside transporter (ENT). This mechanism can contribute to the beneficial effects and to the side effects (dyspnea) of ticagrelor in patients with an acute myocardial infarction.

Objective: To investigate whether ticagrelor increases adenosine receptor stimulation in humans in vivo by ENT inhibition.

Study design: Single centre, double-blinded, randomized placebo-controlled cross-over trial.

Study population: Fourteen healthy male volunteers, aged 18-40 years.

Intervention (if applicable): All subjects will be treated with ticagrelor (180 mg single dose) and with matching placebo in a randomized cross-over design. 2 hours after intake of the drugs, the experiment (vide infra) will be performed.

Main study parameters/endpoints: A venous cannula will be inserted into the antecubital vein of the dominant arm for blood drawing. The brachial artery of the nondominant arm will be cannulated for local drug administration. Forearm blood flow (FBF) will be measured with venous occlusion plethysmography. Subsequently, the FBF response will be measured to 1) administration of adenosine; 2) 2 and 5 minutes of forearm ischemia; 3) administration of dipyridamole, and 4) administration of acetylcholine.

Nature and extent of the burden and risks associated with participation, benefit and group relatedness: A physical examination, electrocardiography and blood sampling will be performed in all participants. All subjects will be treated with ticagrelor (180 mg single dose) or placebo. Potential side effects of ticagrelor include bleeding (epistaxis, skin haematomas, gingival bleeding, gastro-intestinal bleeding, bleeding on puncture side) and dyspnea, but these risks are limited due to the single dose administration and selection of healthy volunteers. Blood will be drawn 5 times during the study (70 ml in total). On both experimental days, a small 27 gauge needle will be inserted into the brachial artery for local drug administration. This can cause a haematoma. The administration of acetylcholine, dipyridamole, and adenosine into the brachial artery has been performed many times previously by our study group, and is considered safe. There is no direct benefit for the participants from this study.

1. INTRODUCTION AND RATIONALE

In the recent *Study of Platelet Inhibition and Patient Outcomes* (PLATO) study, administration of the reversible antagonist of the adenosine diphosphate receptor P2Y12 ticagrelor to patients with an acute myocardial infarction resulted in a striking and unexpected mortality benefit compared to clopidogrel.[1] This finding has instigated the search for an explanation over and above the classical antiplatelet effect of ticagrelor. Another interesting finding in this study was the increased occurrence of dyspnea as an unexpected adverse event in the patients treated with ticagrelor compared to clopidogrel-treated patients.

Given the resemblance of the molecule ticagrelor to the purine nucleoside adenosine, it has been speculated that both the beneficial effects and the side effects of ticagrelor can be mediated by adenosine receptor stimulation.[2] It is well-known that stimulation of adenosine receptors induces various beneficial cardiovascular effects, including vasodilation, inhibition of thrombocyte aggregation, inhibition of inflammation, and increasing resistance against ischemia and reperfusion.[3] In the past years, we and others have demonstrated that various drugs with known cardiovascular protective properties, potentiate adenosine receptor stimulation by interfering with the metabolism of adenosine.[4-9] The metabolism of adenosine is illustrated in Figure 1 (please see end of the protocol).

Adenosine is formed mainly by intra- and extracellular dephosphorylation of AMP, which is catalyzed by 5'-nucleotidase. This enzyme is present in the cytosol and as a membrane-bound extracellular enzyme. In contrast, degradation of adenosine is mainly confined to the intracellular compartment, by adenosine kinase and adenosine deaminase. Consequently, in normal situations, the intracellular concentration is lower than the extracellular concentration, and adenosine transport, which is facilitated diffusion via the equilibrative nucleoside transporter, is directed inwards. Various drugs interfere with this metabolism of adenosine. Dipyridamole increases the extracellular adenosine concentration by inhibition of the ENT transporter.[3] Statins have been shown to promote adenosine receptor stimulation by activation of ecto-5'-nucleotidase activity.[7-9] Metformin facilitates adenosine receptor stimulation, probably by increasing the intracellular formation of adenosine.[5] All these drugs have been reported to limit myocardial infarct size in animal models of acute myocardial infarction, and it is speculated that this is due, at least in part, to adenosine receptor stimulation.[5, 9, 10]

Recent in-vitro studies and studies in animal models have assessed the effects of ticagrelor on the adenosine system. In isolated human erythrocytes, ticagrelor dose-dependently

inhibits the ENT adenosine transporter at concentrations relevant to the clinical situation (IC_{50} 100 nmol/l).[11] This mechanism is similar to the mechanism of dipyridamole. Indeed, in a canine model, administration of both ticagrelor and dipyridamole immediately augmented the reactive hyperemic response in the left coronary artery after brief occlusion, and augmented the vasodilator response to the administration of adenosine into the LAD. In addition, ticagrelor promotes the release of adenosine triphosphate (ATP) from human erythrocytes, which is subsequently degraded to adenosine by membrane bound ecto-nucleotidases.[12] This mechanism of action has been reported with an IC_{50} value of ticagrelor of 14 μmol/l, which is significantly higher than the concentrations reached in patients after oral administration of ticagrelor, and was already observed 5 minutes after the administration of ticagrelor. Both of these mechanisms (augmented extracellular formation of adenosine by increased ATP concentration, and inhibition of cellular uptake of extracellular adenosine via the ENT) can augment adenosine receptor stimulation, which can contribute to the observed beneficial cardiovascular effects of ticagrelor.

In this research proposal, we aim to investigate for the first time in humans in vivo whether ticagrelor promotes adenosine receptor stimulation. In addition, we will explore whether this is due to increased extracellular adenosine formation of adenosine or due to reduced cellular uptake of extracellular adenosine. The results of these studies will provide a possible explanation for the superior efficacy of ticagrelor on cardiovascular outcomes compared to alternative thrombocyte aggregation inhibitors and for the increased occurrence of dyspnea as an adverse effect.

To test our hypotheses, we will use the vasodilator response in the forearm to various stimuli as a well-validated surrogate of adenosine receptor stimulation, as will be explained in more detail in the next section.

2. OBJECTIVES

Primary Objective:

To study whether the administration of ticagrelor augments the forearm vasodilator response to the intra-brachial administration of adenosine. A second primary objective is to investigate whether administration of ticagrelor augments the forearm reactive hyperemic response to 2 and 5 minutes of forearm ischemia.

Secondary Objective(s):

- To investigate whether the administration of ticagrelor potentiates the forearm vasodilator response to the intrabrachial administration of dipyridamole.
- To study whether ticagrelor inhibits the *ex vivo* adenosine uptake in isolated erythrocytes.

3. STUDY DESIGN

In this randomized placebo-controlled double-blinded cross-over study, we will test the following hypotheses:

1. Treatment with ticagrelor inhibits the ENT transporter on circulating erythrocytes.
2. Treatment with ticagrelor augments the vasodilator response to the intrabrachial administration of adenosine due to inhibition of the ENT transporter.
3. Treatment with ticagrelor augments the reactive hyperemia that occurs in response to forearm ischemia by preventing the cellular uptake of endogenous adenosine.
4. Treatment with ticagrelor does not potentiate the vasodilator response to the administration of the ENT inhibitor dipyridamole and might even attenuate this vasodilator response because there is less potential for additional ENT inhibition by dipyridamole if ticagrelor has already induced ENT inhibition.

The design of the study is depicted in Figure 2 (please see end of the protocol). This design is comparable to the design we used in previous studies. [4, 6, 7, 13] In brief, fourteen healthy male volunteers (age 18-40 years) will be asked to participate. After signing for informed consent, history taking, a physical examination, and electrocardiography will be performed. We would like te refer to paragraph 4.2 and 4.3 for the in-, and exclusion criteria.

After inclusion, each subject will participate in two experiments, separated by 2 weeks. Two hours before starting the administration of adenosine into the brachial artery, the subjects will

take a single oral dose of either ticagrelor (180 mg single dose) or placebo in a randomized, double-blinded cross-over design (see figure 2). The justification of the dose and timing of administration is given in chapter 6.

On both days, the subjects will take the study medication under supervision. The subjects will be studied in supine position after an overnight fast and at least 24 h of caffeine abstinence (since caffeine is an adenosine receptor antagonist). Experiments are performed in a temperature-controlled room (24 °C) in the morning after an overnight fast. Before intake of the study medication, a venous cannula will be inserted into the antecubital vein of the dominant arm for blood drawing. Blood will be drawn (10 ml each time) before intake of the study medication for the determination of the plasma caffeine and ticagrelor concentration. In addition, blood will be drawn immediately before the administration of adenosine for determination of the ticagrelor concentration, and to determine the ex vivo ENT transport characteristics in isolated erythrocytes, as previously described[14]. Finally, 10 ml of blood will be drawn immediately before the administration of dipyridamole and acetylcholine into the brachial artery and before forearm ischemia for the determination of the plasma ticagrelor concentration. From every timepoint, serum will be kept in the freezer to be able to also measure the major circulating active metabolite of ticagrelor AR-C124910XX, if this is deemed to be of additional value.

In addition, A 27-gauge needle (B. Braun Medical B.V.) is inserted into the brachial artery of the nondominant arm for intra-arterial drug administration. In both arms, forearm blood flow (FBF) is measured simultaneously with venous occlusion plethysmography using mercury-in-silastic-strain gauges and occluded hand circulation as described previously.[6] Thirty minutes after cannulation of the brachial artery, normal saline is infused with concomitant measurement of baseline FBF for 5 min (figure 2). Subsequently, four experiments will be performed, which are all separated by 30 minutes of wash-out.

1. FBF will be measured during the administration of increasing dosages of adenosine into the brachial artery. These dosages are similar to a previous study of our group in which we demonstrated that the vasodilator response was potentiated by concomitant administration of the ENT inhibitor dipyridamole.[13] We hypothesize that during treatment with ticagrelor, the adenosine-induced vasodilation is potentiated due to effective inhibition of adenosine uptake via the ENT transporter.
2. After 30 minutes of washout, the FBF will be measured after 2 and 5 minutes of forearm ischemia ('post-occlusive reactive hyperemia'). Forearm ischemia will be induced by inflation of an upper arm cuff to 200 mmHg, as described previously.[7] Between the last flow measurement and subsequent occlusion of the forearm, 5 min

of extra reperfusion will allow the hand circulation to recover from the wrist cuff inflations. We have previously demonstrated that the PORH after 2 and 5 minutes of ischemia is potentiated by statins (by increasing the extracellular formation of adenosine)[7] and by dipyridamole (by inhibition of the ENT transporter).[6] We hypothesize that ticagrelor augments the PORH. Most probably this is due to ENT inhibition, but increased extracellular formation of adenosine due to higher ATP release from erythrocytes could also contribute. Interestingly, these two studies can already differentiate between these two possible mechanisms: if ticagrelor solely functions as ENT inhibitor, than the vasodilator response in experiment 1 and 2 is potentiated, whereas when ticagrelor only increases the extracellular formation of adenosine, than the vasodilator response is solely augmented in experiment 2.

3. After 30 minutes of recovery, the vasodilator response to the administration of dipyridamole into the brachial artery is measured. We will use the dipyridamole dose that we have used in previous studies.[4] Dipyridamole increases FBF by ENT inhibition and the subsequent rise of the extracellular adenosine concentration. When ticagrelor affects the adenosine system only by ENT inhibition, the vasodilator response to dipyridamole will not be potentiated. In this situation, the dipyridamole-induced vasodilation might even be attenuated by ticagrelor, because there is less opportunity for additional ENT inhibition by dipyridamole.
4. Finally, after 30 minutes of washout, the forearm vasodilator response to the administration of acetylcholine will be recorded as a negative control-experiment. Acetylcholine induces endothelium-dependent but adenosine-independent vasodilation. By showing that acetylcholine-induced vasodilation is not affected by ticagrelor, we exclude non-specific effects of ticagrelor on the endothelium or vascular smooth muscle cell.

In addition to the FBF experiment described above, the effect of ticagrelor on ENT transport capacity will be investigated in an ex-vivo experiment. From all volunteers, ten ml of blood will be collected by venipuncture before the intake of ticagrelor/placebo and immediately before starting the experiment (i.e. approximately 2 hours after intake of the last dose of ticagrelor). Erythrocytes and lymphocytes will be isolated as described previously. [7, 14] Subsequently, the ENT activity will be measured in isolated erythrocytes by studying the uptake of adenosine and uridine (uridine is used because it is also taken up via the ENT, but it is not metabolized in the erythrocyte, in contrast to adenosine), as described previously. [14]. In the lymphocytes, we will measure the activity of ecto-5'-nucleotidase by quantifying the conversion of 1,N^6-ethenoadenosine 5'-AMP to 1,N^6-ethoenoadenosine in the presence and absence of a specific inhibitor of ecto-5'-nucleotidase, to study whether ticagrelor also affects

ecto-5'-nucleotidase activity. [7] Also, immediately before each experiment (together with the blood for ENT inhibition assays), 3 ml of blood will be sampled for the determination of the circulation caffeine concentration to check compliance with caffeine abstinence. Subjects with a circulating caffeine concentration > 1.0 mg/l will be excluded from analyses.

4. STUDY POPULATION

4.1 Population (base)

We will recruit 14 healthy male volunteers, aged 18-40 years. We will only include male patients to exclude any potential effect of circulating hormones on our outcome parameters, and to exclude an effect of ticagrelor on foetal development in case of pregnancy.

The subjects will be recruited by advertisements placed throughout the university campus, the university sports centre, student flats, and placed on the university Intranet site.

Given our experience with previous studies with healthy male subjects, we do not expect any problems to recruit the 14 subjects for this study.

4.2 Inclusion criteria

In order to be eligible to participate in this study, a subject must meet all of the following criteria:

- Male sex
- Age 18-40 years
- Healthy
- Written informed consent

4.3 Exclusion criteria

A potential subject who meets any of the following criteria will be excluded from participation in this study:

- Smoking
- Hypertension (Blood pressure >140 mmHg and/or >90 mmHg – SBP/DBP-)
- Diabetes Mellitus (fasting glucose > 7.0 mmol/L or random > 11.0 mmol/L)
- History of any cardiovascular disease
- History of chronic obstructive pulmonary disease (COPD) or asthma
- Bleeding tendency
- Concomitant use of medication
- Renal dysfunction (MDRD < 60 ml/min)
- Liver enzyme abnormalities (ALAT > twice upper limit of normality)
- Thrombocytopenia ($<150*10^9$/ml)
- Second/third degree AV-block on electrocardiography

4.4 Sample size calculation

Several previous studies give an indication of the magnitude of the effect of ticagrelor on adenosine-induced vasodilation or post-occlusive reactive hyperemia. In a recent study in dogs, the effect of systemic administration of ticagrelor on the vasodilator effect of adenosine in the left coronary artery was investigated. In this study, adenosine-induced vasodilation was augmented by 150% in the ticagrelor-treated group [11]. We have previously shown in healthy humans that concomitant administration of dipyridamole into the brachial artery augmented the vasodilator response to the administration of adenosine fivefold [13]. Our own research group has previously studied the effect of the ENT-inhibitor dipyridamole on the forearm post-occlusive reactive hyperemia in healthy subjects: administration of dipyridamole increased this flow from 3.2 to 4.7 ml/min after 2 minutes of forearm ischemia and from 7.3 to 9.5 ml/min after 5 minutes of ischemia (increase with 145% and 130%) [6].

The power analysis of this study has been performed by a statistician of the department of Epidemiology, Biostatistics, and HTA of the Radboud University Nijmegen Medical Centre (prof. G. Borm). In previous studies from our own group, we found that the vasodilator response to the intrabrachial administration of adenosine averages 2.8±0.6, 4.4±1.0, 9.0±1.7 ml/min per dl of forearm volume for 0.5, 1.5, and 5.0 μg/min/dl, respectively (mean±SEM, n=8). The pooled CV is 0.6, so after log transformation the SD averaged 0.55. In our study measurements are repeated within the same subject, and we assume a correlation between any two measurements of 0.7. We expect that a (relative) difference between the treatments is independent of the adenosine dose. Hence, a linear mixed model will be used, with fixed factors treatment (ticagrelor vs placebo), adenosine dose, and period. This analysis will have approximately the same power as a one sample t-test on the contrast (log_flow $_{dose\_1\_tica}$ + log_ flow $_{dose\_2\_tica}$ +log_ flow $_{dose\_3\_tica}$) /3 - (log_ flow $_{dose\_1\_placebo}$+ log_ flow $_{dose\_2\_placebo}$ +log_ flow $_{dose\_3\_placebo}$)/3. Based on a correlation of 0.7 between the measurements for all doses and time points and an sd of 0.55, the sd of the contrast is sqrt(2/3 – 2/3*0.7)*0.55=0.25. As a result, 13 evaluable subjects are needed. to demonstrate an augmentation of adenosine-induced vasodilation with 1.25 (ie a 25% increase) with a power of 80% and a two-sided alpha of 0.05. To account for drop-out, we aim to include 14 healthy volunteers,

5. TREATMENT OF SUBJECTS

5.1 Investigational product/treatment

All subjects will receive a single oral dose of ticagrelor (180 mg) and placebo in a randomized double-blinded cross-over design.

5.2 Use of co-intervention

No co-medication is allowed. The experiments will be performed in the morning after an overnight fast and at least 24 hours of caffeine abstinence, because caffeine is an effective adenosine receptor antagonist. Also, subjects have to abstain from alcohol for 24 hours.

5.3 Escape medication

Not applicable.

6. INVESTIGATIONAL PRODUCT

6.1 Name and description of investigational product

Subjects will receive either a single dose of ticagrelor (180 mg) of matching placebo. Ticagrelor is a selective reversible adenosine diphosphate (ADP) receptor antagonist acting on the P2Y12 ADP-receptor that can prevent ADP-mediated platelet activation and aggregation. Ticagrelor is orally active, and reversibly interacts with the platelet P2Y12 ADP-receptor. It is indicated, co-administered with acetylsalicylic acid (ASA), for the prevention of atherothrombotic events in adult patients with Acute Coronary Syndromes (unstable angina, non ST elevation Myocardial Infarction [NSTEMI] or ST elevation Myocardial Infarction [STEMI]); including patients managed medically, and those who are managed with percutaneous coronary intervention (PCI) or coronary artery by-pass grafting (CABG).

6.2 Summary of findings from non-clinical studies

We would like to refer to page 16 of the Summary of Products Characteristics of ticagrelor. In addition, in-vitro studies and studies in animal models have assessed the effects of ticagrelor on the adenosine system. In isolated human erythrocytes, ticagrelor dose-dependently inhibits the ENT adenosine transporter at concentrations relevant to the clinical situation (IC_{50} 100 nmol/l).[11] This mechanism is similar to the mechanism of dipyridamole. Indeed, in a canine model, administration of both ticagrelor and dipyridamole immediately augmented the reactive hyperemic response in the left coronary artery after brief occlusion, and augmented the vasodilator response to the administration of adenosine into the LAD. In addition, ticagrelor promotes the release of adenosine triphosphate (ATP) from human erythrocytes, which is subsequently degraded to adenosine by membrane bound ecto-nucleotidases.[12] This mechanism of action has been reported with an IC_{50} value of ticagrelor of 14 µmol/l, which is much higher than the concentrations reached in patients after oral administration of ticagrelor, and was already observed 5 minutes after the administration of ticagrelor. Both of these mechanisms (augmented extracellular formation of adenosine by increased ATP concentration, and inhibition of cellular uptake of extracellular adenosine via the ENT) can augment adenosine receptor stimulation, which can contribute to the observed beneficial cardiovascular effects of ticagrelor.

6.3 Summary of findings from clinical studies

In the PLATO study, a landmark study in 18.624 patients with an acute myocardial infarction, treatment with ticagrelor (180 mg loading dose, followed by 90 mg

bid for one year) reduced the incidence of the primary endpoint (composite of vascular death, myocardial infarction, or stroke) from 11.7% to 9.8% compared with treatment with clopidogrel.

6.4 Summary of known and potential risks and benefits

There are no direct benefits from treatment for the subjects participating in this study. For the side-effects, we would like to refer to the SPC of ticagrelor. The two main potential side effects are bleeding and dyspnea.

In the PLATO trial, ticagrelor and clopidogrel did not differ in Major Fatal/Life-threatening bleeding, total Major bleeding, TIMI Major bleeding, or TIMI Minor bleeding. However, more combined Major + Minor bleeding occurred with ticagrelor compared with clopidogrel. Few patients in PLATO had fatal bleeds: 20 (0.2%) for ticagrelor and 23 (0.3%) for clopidogrel. It has to be realized however, that PLATO concerned patients who were treated concomitantly with aspirin, and who were treated with PCI or CABG. Our study concerns healthy volunteers without a bleeding tendency and with normal circulating platelets. Moreover, ticagrelor is administered in a single dose. Given the half life of 7 hours, inhibition of thrombocyte aggregation is present only for one day.

A second potential side effect is dyspnea. In the SPC of ticagrelor, it is mentioned that "Dyspnoea adverse reactions (ADRs) (dyspnoea, dyspnoea at rest, dyspnoea exertional, dyspnoea paroxysmal nocturnal and nocturnal dyspnoea), when combined, was reported by 13.8% of patients treated with ticagrelor and by 7.8% of patients treated with clopidogrel. In 2.2% of patients taking ticagrelor and by 0.6% taking clopidogrel investigators considered the dyspnoea causally related to treatment in the PLATO study and few were serious (0.14% ticagrelor; 0.02% clopidogrel). Most reported symptoms of dyspnoea were mild to moderate in intensity, and most were reported as a single episode early after starting treatment. Compared with clopidogrel, patients with asthma/COPD treated with ticagrelor may have an increased risk of experiencing non-serious dyspnoea (3.29% ticagrelor versus 0.53% clopidogrel) and serious dyspnoea (0.38% ticagrelor versus 0.00% clopidogrel). In absolute terms, this risk was higher than in the overall PLATO population. Ticagrelor should be used with caution in patients with history of asthma and/or COPD. ". In the current study, we will exclude subjects with a history of asthma or COPD.

There are several studies in which ticagrelor is given to healthy male subjects. E.g. in the study by Teng et al, 24 healthy male volunteers were treated with ticagrelor (90 mg bid for 7 days) with or without concomitant treatment with simvastatin or atorvastatin.[15] Two volunteers receiving ticagrelor and atorvastatin had ecchymoses (classified as minor bleeding), which were considered treatment-related. Three volunteers

receiving ticagrelor and simvastatin had dyspnoea (two events considered treatment related) and four experienced one minor bleeding event each (epistaxis, cephalhaematoma, haematuria [considered treatment-related], and ecchymosis). In a recent study by Butler et al, 24 healthy male volunteers were treated with ticagrelor for 5 days (90 mg bid) [16]. In this study six treatment-related adverse events occurred in 3 (13%) volunteers while receiving ticagrelor; constipation, nausea, vomiting, headache, conjunctival hemorrhage, and rash. One volunteer had one treatment-related adverse event (headache) with placebo administration. All adverse events were mild, except for the rash which was moderate in intensity. All adverse events resolved during the study, except for the rash and a mild-intensity body tinea (not treatment related). In this study, there were no reports of dyspnea. The mild conjunctival hemorrhage started at 23 h post dosing with ticagrelor and took approximately 5 days to resolve. In an escalating single-dose study in 13 healthy volunteers, purpura were the only reported side effect with an incidence of 55% following placebo, and 63, 38, and 29% after 200, 300, and 400 mg of ticagrelor, respectively. All purpura were mild. [17].

6.5 Description and justification of route of administration and dosage

Ticagrelor is administered orally in a single dose of 180 mg. This dosage is registered as a loading dose in patients with an acute myocardial infarction. We chose to perform the experiment after a single dose, because we expect that the effect of ticagrelor on the adenosine system occurs instantaneously after a single dose. This is based on the observation that the ENT is inhibited, and adenosine-induced vasodilation is potentiated immediately after the administration of ticagrelor. [11]. Also the effect on thrombocyte aggregation is rapid: after a loading dose of 180 mg, the mean inhibition of platelet aggregation is 41% after 0.5 hours, with a maximum effect of 89% after 2-4 hours. The T_{max} of ticagrelor after oral dosing is 1.5 hours, and 2.5 hours with the major circulating active metabolite AR-C124910XX (SPC Ticagrelor). Based on these parameters, we will administer the ticagrelor 2 hours before initiation of the experiment.

6.6 Dosages, dosage modifications and method of administration

Please see previous section.

6.7 Preparation and labelling of Investigational Medicinal Product

Study medication will be prepared by the “Apotheek Haagse Ziekenhuizen” te Den Haag. The drugs will be shipped to the department of Clinical Pharmacy of the

Radboud University Nijmegen Medical Centre, and further distributed to the Clinical Research Centre Nijmegen, where the drugs will be stocked under GMP-conditions.

6.8 Drug accountability

The department of Clinical Pharmacy of the Radboud University Medical Centre will provide the ticagrelor and placebo used in this trial. The products are transported to the Clinical Research Centre Nijmegen at the RUNMC and stored there under GMP conditions. All tablets will be ingested under supervision.

7. NON-INVESTIGATIONAL PRODUCT

7.1 Name and description of non-investigational product(s)

During the experiments, we will administer several agents into the brachial artery:

1. adenosine
2. dipyridamole
3. acetylcholine

All these compounds are given into the brachial artery to ensure a high local concentration in the forearm vascular bed, whereas the systemic circulating concentration is very low to prevent any systemic effects. All these compounds have been used, in the same dosages, in many previous studies from our department.

7.2 Summary of findings from non-clinical studies

We kindly refer to the SPC's of the three drugs, which are attached.

7.3 Summary of findings from clinical studies

For details, see SPC text. Adenosine and dipyridamole are currently registered for myocardial stress testing. The dose we use in our study, however, is much lower than the dosages used for these purposes. Finally, acetylcholine is indicated for injection into the anterior eye chamber to induce rapid dilation of the pupil.

7.4 Summary of known and potential risks and benefits

We will administer all drugs into the brachial artery. This approach guarantees an effective concentration into the forearm vascular bed, which induces rapid forearm skeletal muscle vasodilation. In the past decades, we have given these drugs in these concentration very often into the brachial artery of healthy subjects, and we have never seen any side-effects. Therefore, we are convinced that the use of these compounds is without any risks.

7.5 Description and justification of route of administration and dosage

We will administer all drugs into the brachial artery. This approach guarantees an effective concentration into the forearm vascular bed, which induces rapid forearm skeletal muscle vasodilation. In the past decades, we have given these drugs in these concentration very often into the brachial artery of healthy subjects, and we have never seen any side-effects. Therefore, we are convinced that the use of these compounds is without any risks.

The various dosages for each drug are based on our own previous studies with these drugs:

1. Adenosine: 0.15, 0.5, 1.5 and 5.0 μg/min per dl of forearm volume for 5 minutes for each concentration. This dosage is based on a previous study from our group (e.g. [13]: these dosages give moderate forearm vasodilation (forearm blood flow from 2.5

to 7 ml/min), which can be potentiated by ENT inhibition (such as dipyridamole and possibly ticagrelor), without inducing any systemic effects.

2. Dipyridamole: 30 and 100 µg/min/dl. This dosage is based on a previous study from our group, in which we have reported that these dosages give moderate forearm vasodilation, without inducing any systemic effects. [4].
3. Acetylcholine will be given in a dose of 0.5 and 2.0 µg/min/dl. This dosage has previously been used in our group and induces a moderate increase in forearm blood flow during infusion (from 3.0 to 6.0 and 8.7 ml/min/dl) [18].

7.6 Dosages, dosage modifications and method of administration

Please see above

7.7 Preparation and labelling of Non Investigational Medicinal Product

All products will be provided by the department of Clinical Pharmacy of the Radboud University Nijmegen Medical Centre and stocked under GMP conditions at the Clinical Research Centre Nijmegen. The final solutions will be prepared by the investigator on the day of the experiment using saline.

7.8 Drug accountability

Please see above

8. METHODS

8.1 Study parameters/endpoints

8.1.1 Main study parameter/endpoint

Forearm blood flow response to the intrabrachial administration of adenosine. ~~and to 2 and 5 minutes of forearm ischemia (i.e. post-occlusive reactive hyperemia).~~

8.1.2 Secondary study parameters/endpoints (if applicable)

Forearm blood flow response to ~~and to~~ 2 and 5 minutes of forearm ischemia (i.e. post-occlusive reactive hyperemia).and to the intrabrachial administration of dipyridamole.

Transport characteristics of the ENT transporter, determined *ex vivo* on isolated erythrocytes.

8.1.3 Other study parameters (if applicable)

Not applicable

8.2 Randomisation, blinding and treatment allocation

The study is a double-blinded randomized placebo-controlled cross-over study. Randomisation will be performed by the Apotheek Haagse Ziekenhuizen. The randomisation code will be kept by the department of Clinical Pharmacy of the Radboud University Nijmegen Medical Centre.

8.3 Study procedures

We would like to refer to chapter 3 (study design) in which the study protocol is described in detail.

In summary:

- Medical screening: history taking, physical examination, electrocardiogram, venous puncture of three 3 ml-vacutainers for the determination of creatinin, ALAT, thrombocytes, cholesterol and glucose.
- Experiment:
  - Insertion of venous cannula into the antecubital vein of the dominant arm for blood drawing (20 ml before administration of the study drug for ex vivo ENT transport measurements, and measurement of the circulating caffeine and ticagrelor concentration; 20 ml immediately before the administration of adenosine for measurement of ticagrelor and ENT transport characteristics, and 10 ml before the administration of dipyridamole, the administration of acetylcholine, and before forearm ischemia for determination of the ticagrelor concentration.)

- Insertion of 27 Gauge needle into the brachial artery of the nondominant arm for drug administration of adenosine, dipyridamole, and acetylcholine (please see previous paragraph for dosages).
- Venous occlusion plethysmography of the forearm to determine the forearm blood flow responses to the administration of these drugs, and to 2 and 5 minutes of forearm ischemia.

8.4 Withdrawal of individual subjects

Subjects can leave the study at any time for any reason if they wish to do so without any consequences. The investigator can decide to withdraw a subject from the study for urgent medical reasons.

8.4.1 Specific criteria for withdrawal (if applicable)

Not applicable

8.5 Replacement of individual subjects after withdrawal

In this study, we will include one subject more than the number of subjects needed according to our power analyses to account for a drop-out. In case that more than one subject will withdraw from the study, we will replace the subject with another subject, with a maximum of 2 additional volunteers.

8.6 Follow-up of subjects withdrawn from treatment

A participant who decides to withdraw from the trial will be invited to an exit-interview concerning their withdrawal.

8.7 Premature termination of the study

Not applicable.

9. SAFETY REPORTING

9.1 Section 10 WMO event

In accordance to section 10, subsection 1, of the WMO, the investigator will inform the subjects and the reviewing accredited METC if anything occurs, on the basis of which it appears that the disadvantages of participation may be significantly greater than was foreseen in the research proposal. The study will be suspended pending further review by the accredited METC, except insofar as suspension would jeopardise the subjects' health. The investigator will take care that all subjects are kept informed.

9.2 AEs, SAEs and SUSARs

9.2.1 Adverse events (AEs)

Adverse events are defined as any undesirable experience occurring to a subject during the study, whether or not considered related to the investigational product / the experimental intervention. All adverse events reported spontaneously by the subject or observed by the investigator or his staff will be recorded.

9.2.2 Serious adverse events (SAEs)

A serious adverse event is any untoward medical occurrence or effect that at any dose:

- results in death;
- is life threatening (at the time of the event);
- requires hospitalisation or prolongation of existing inpatients' hospitalisation;
- results in persistent or significant disability or incapacity;
- is a congenital anomaly or birth defect;
- Any other important medical event that may not result in death, be life threatening, or require hospitalization, may be considered a serious adverse experience when, based upon appropriate medical judgement, the event may jeopardize the subject or may require an intervention to prevent one of the outcomes listed above.

All serious adverse events will be reported to the sponsor of this trial: Prof. F. Russel, head of the department of Pharmacology and Toxicology. He delegates the appropriate handling of these events to the coordinating and principle investigators of this trial: Dr. G. A. Rongen and Dr. N.P. Riksen, Further report of these adverse events will be performed according to GCP as outlined below.

The sponsor will report the SAEs through the web portal *ToetsingOnline* to the accredited METC that approved the protocol, within 15 days after the sponsor has first knowledge of the serious adverse reactions.

SAEs that result in death or are life threatening should be reported expedited. The expedited reporting will occur not later than 7 days after the responsible investigator has first knowledge of the adverse reaction. This is for a preliminary report with another 8 days for completion of the report.

9.2.3 Suspected unexpected serious adverse reactions (SUSARs)

Adverse reactions are all untoward and unintended responses to an investigational product related to any dose administered.

Unexpected adverse reactions are SUSARs if the following three conditions are met:

1. the event must be serious (see chapter 9.2.2);
2. there must be a certain degree of probability that the event is a harmful and an undesirable reaction to the medicinal product under investigation, regardless of the administered dose;
3. the adverse reaction must be unexpected, that is to say, the nature and severity of the adverse reaction are not in agreement with the product information as recorded in:
   - Summary of Product Characteristics (SPC) for an authorised medicinal product;
   - Investigator’s Brochure for an unauthorised medicinal product.

The sponsor will report expedited the following SUSARs through the web portal *ToetsingOnline* to the METC:

- SUSARs that have arisen in the clinical trial that was assessed by the METC;
- SUSARs that have arisen in other clinical trials of the same sponsor and with the same medicinal product, and that could have consequences for the safety of the subjects involved in the clinical trial that was assessed by the METC.

The remaining SUSARs are recorded in an overview list (line-listing) that will be submitted once every half year to the METC. This line-listing provides an overview of all SUSARs from the study medicine, accompanied by a brief report highlighting the main points of concern.

The expedited reporting of SUSARs through the web portal ToetsingOnline is sufficient as notification to the competent authority.

The sponsor will report expedited all SUSARs to the competent authorities in other Member States, according to the requirements of the Member States.

The expedited reporting will occur not later than 15 days after the sponsor has first knowledge of the adverse reactions. For fatal or life threatening cases the term will be maximal 7 days for a preliminary report with another 8 days for completion of the report.

If a SAE or SUSAR occurs and there is the need to know whether a patient was treated with ticagrelor or placebo, the pharmacist on call can be contacted (24 hours a day, 7 days a week) and he/she will be able to break the blinding code for that particular participant.

9.3 Annual safety report

In addition to the expedited reporting of SUSARs, the sponsor will submit, once a year throughout the clinical trial, a safety report to the accredited METC, competent authority, and competent authorities of the concerned Member States.

This safety report consists of:

- a list of all suspected (unexpected or expected) serious adverse reactions, along with an aggregated summary table of all reported serious adverse reactions, ordered by organ system, per study;
- a report concerning the safety of the subjects, consisting of a complete safety analysis and an evaluation of the balance between the efficacy and the harmfulness of the medicine under investigation.

9.4 Follow-up of adverse events

All AEs will be followed until they have abated, or until a stable situation has been reached. Depending on the event, follow up may require additional tests or medical procedures as indicated, and/or referral to the general physician or a medical specialist.

SAEs need to be reported till end of study within the Netherlands, as defined in the protocol

9.5 Data Safety Monitoring Board (DSMB) / Safety Committee

Not applicable

10. STATISTICAL ANALYSIS

10.1 Primary study parameter(s)

The forearm blood flow response to the administration of adenosine and to the 2 and 5 minutes of forearm ischemia after treatment with ticagrelor of placebo will be compared using a mixed-model analysis. A p-value of < 0.05 will be considered to be statistically significant.

10.2 Secondary study parameter(s)

The forearm blood flow response to the administration of dipyridamole and acetylcholine after treatment with ticagrelor of placebo will be compared using a mixed-model analysis.

For the ENT transporter on erythrocytes, the Vmax and Km value will be determined for each subject. These values will be compared between the ticagrelor and placebo treatment period with a paired Student's t-test.

10.3 Other study parameters

Not applicable

10.4 Interim analysis (if applicable)

Not applicable

11. ETHICAL CONSIDERATIONS

11.1 Regulation statement

This study will be conducted according to the principles of the Declaration of Helsinki (version: April 2008) and in accordance with the Medical Research Involving Human Subjects Act (WMO) and other guidelines and regulations.

11.2 Recruitment and consent

Healthy volunteers will be recruited at the University Medical Centre Nijmegen (please see chapter 4). Informed consent will be achieved by a ‘participation information letter’ and potential participants will be given the opportunity to address their questions to the investigator. Participation is only possible after written informed consent. Please find the patient information letter and informed consent form as separate documents.

11.3 Objection by minors or incapacitated subjects (if applicable)

Not applicable

11.4 Benefits and risks assessment, group relatedness

The study does not concern minors or incapacitated subjects. The subjects participating in this study do not benefit from participation. The risks of participation are low (please see paragraph 6.4). The results of our study increase the knowledge about the mechanism of action of ticagrelor. Potentially, this knowledge can be used in the future to optimize clinical benefit of ticagrelor or limit side-effects. Therefore, to our opinion, the potential benefit from this study outweighs the risk for the participating subjects.

11.5 Compensation for injury

The sponsor/investigator has a liability insurance which is in accordance with article 7, subsection 6 of the WMO.

The sponsor (also) has an insurance which is in accordance with the legal requirements in the Netherlands (Article 7 WMO and the Measure regarding Compulsory Insurance for Clinical Research in Humans of 23th June 2003). This insurance provides cover for damage to research subjects through injury or death caused by the study.

1. € 450.000,-- (i.e. four hundred and fifty thousand Euro) for death or injury for each subject who participates in the Research;

2. € 3.500.000,-- (i.e. three million five hundred thousand Euro) for death or injury for all subjects who participate in the Research;
3. € 5.000.000,-- (i.e. five million Euro) for the total damage incurred by the organisation for all damage disclosed by scientific research for the Sponsor as 'verrichter' in the meaning of said Act in each year of insurance coverage.

The insurance applies to the damage that becomes apparent during the study or within 4 years after the end of the study.

11.6 Incentives (if applicable)

The subjects will receive 200 euros as a compensation for participation in the study. Should a subject withdraw after the first experiment, 100 euros will be paid.

12. ADMINISTRATIVE ASPECTS, MONITORING AND PUBLICATION

12.1 Handling and storage of data and documents

All research outcomes will be archived in the personal medical file of each participant. This medical file will not leave the Radboud University Nijmegen Medical Centre. This source document can only be viewed by trial monitors and investigators involved. The following data will be archived in the source document: Copy of signed informed consent form, EKG, Subject study code, each visit date and all study outcomes (laboratory) and possible adverse events. In addition to the source document, a case report form (CRF) will be completed. The CRF is anonymised (only contains the subject study code) and may leave the hospital for data management or monitoring purposes. A copy of this CRF remains at the RUNMC for 15 years.

The following data will be archived in the CRF: Subject code; date, all study outcomes (laboratory) and possible adverse events, (serious) adverse events. A subject code log will be archived in the trial master file. Furthermore we will file the drug accountability forms in the trial master file together with the approved version of the protocol, correspondence with the METC and CVs of all involved investigators.

12.2 Monitoring and Quality Assurance

According to the NFU publication “kwaliteitsborging van mensgebonden onderzoek”, the participants of our study have a <u>small change</u> of <u>moderate risk</u>. However, we believe that given the complexity of the experiment, a <u>moderate intensive monitoring</u> according to the NFU guideline, is warranted. Monitoring will be performed by a Clinical Research Associate of the Clinical Research Centre Nijmegen.

According to the NFU guidelines, three visits will be performed (initiation, one after inclusion of half of the participants, and one closure visit). Informed consent, SAE’s and SUSAR’s will be checked for all participants. In-, and exclusion criteria will be checked for the first 7 participants. A complete source data verification will be performed for 4 subjects. Please find attached at the end of the protocol a separate monitoring plan.

12.3 Amendments

A ‘substantial amendment’ is defined as an amendment to the terms of the METC application, or to the protocol or any other supporting documentation, that is likely to affect to a significant degree:

- the safety or physical or mental integrity of the subjects of the trial;
- the scientific value of the trial;
- the conduct or management of the trial; or

- the quality or safety of any intervention used in the trial.

All substantial amendments will be notified to the METC and to the competent authority.

Non-substantial amendments will not be notified to the accredited METC and the competent authority, but will be recorded and filed by the sponsor.

12.4 Annual progress report

The sponsor/investigator will submit a summary of the progress of the trial to the accredited METC once a year. Information will be provided on the date of inclusion of the first subject, numbers of subjects included and numbers of subjects that have completed the trial, serious adverse events/ serious adverse reactions, other problems, and amendments.

12.5 End of study report

The sponsor will notify the accredited METC and the competent authority of the end of the study within a period of 90 days. The end of the study is defined as the last patient's last visit.

In case the study is ended prematurely, the sponsor will notify the accredited METC and the competent authority within 15 days, including the reasons for the premature termination.

Within one year after the end of the study, the investigator/sponsor will submit a final study report with the results of the study, including any publications/abstracts of the study, to the accredited METC and the Competent Authority.

12.6 Public disclosure and publication policy

Please see paragraphs 4.1 and 4.2 of the contract between AstraZeneca and dr. M. Gomes. Before initiation of the study, the study details will be published on ClinicalTrials.gov. After completion of the study, the results will be submitted to a scientific journal for publication.

13. STRUCTURED RISK ANALYSIS

13.1 Potential issues of concern

a. Level of knowledge about mechanism of action

The mechanism of action of ticagrelor is well known and described in detail in the SPC which is attached. In this study, we aim to investigate whether ticagrelor also modulates the kinetics of the endogenous purine nucleoside adenosine. This hypothesis is based on previous in vitro experiments and animal studies which are described in detail in chapter 1 of this protocol.

b. Previous exposure of human beings with the test product(s) and/or products with a similar biological mechanism

Ticagrelor is registered for the treatment of patients with an acute myocardial infarction, as described in detail in the SPC.

c. Can the primary or secondary mechanism be induced in animals and/or in *ex-vivo* human cell material?

The aim of our study is to translate previous in vitro findings and findings in animal studies to the human in vivo situation. Therefore, our study has to be conducted in humans.

d. Selectivity of the mechanism to target tissue in animals and/or human beings

Please see SPC.

e. Analysis of potential effect

The primary endpoint of the study is the forearm blood flow response to the administration of adenosine into the brachial artery. This is detected by venous occlusion plethysmography of the forearm.

f. Pharmacokinetic considerations

We would like to refer to the SPC and to paragraph 6.5 of this protocol for a detailed description of the pharmacokinetic properties of ticagrelor.

g. Study population

The study is performed in healthy male volunteers.

h. Interaction with other products

Not applicable

i. Predictability of effect

Not applicable

j. Can effects be managed?

Not applicable

13.2 Synthesis

Given the administration of a single dose of ticagrelor to healthy male subjects without bleeding problems and with normal circulating thrombocytes, and given the very small 27 gauge needle used for intrabrachial administration of adenosine, dipyridamole, and acetylcholine, we think that the potential risks for the volunteers are low. Also, the methods used (venous blood drawing, venous occlusion plethysmography, and insertion of a cannula into the brachial artery) are well-established and have been performed very often by our research group, without any serious events.

14. REFERENCES

Reference List

(1) Wallentin L, Becker RC, Budaj A et al. Ticagrelor versus clopidogrel in patients with acute coronary syndromes. *N Engl J Med* 2009 September 10;361(11):1045-57.

(2) Serebruany VL. Adenosine release: a potential explanation for the benefits of ticagrelor in the PLATelet inhibition and clinical outcomes trial? *Am Heart J* 2011 January;161(1):1-4.

(3) Riksen NP, Rongen GA. Targeting adenosine receptors in the development of cardiovascular therapeutics. *Expert Rev Clin Pharmacol* 2012 March;5(2):199-218.

(4) Riksen NP, Barrera P, Van den Broek PH, van RP, Smits P, Rongen G. Methotrexate modulates the kinetics of adenosine in humans in vivo. *Ann Rheum Dis* 2005 November 24.

(5) Paiva M, Riksen NP, Davidson SM et al. Metformin prevents myocardial reperfusion injury by activating the adenosine receptor. *J Cardiovasc Pharmacol* 2009 May;53(5):373-8.

(6) Meijer P, Wouters CW, Van den Broek PH et al. Dipyridamole enhances ischaemia-induced reactive hyperaemia by increased adenosine receptor stimulation. *Br J Pharmacol* 2008 March;153(6):1169-76.

(7) Meijer P, Wouters CW, Van den Broek PH et al. Upregulation of ecto-5'-nucleotidase by rosuvastatin increases the vasodilator response to ischemia. *Hypertension* 2010 October;56(4):722-7.

(8) Meijer P, Oyen WJ, Dekker D et al. Rosuvastatin increases extracellular adenosine formation in humans in vivo: a new perspective on cardiovascular protection. *Arterioscler Thromb Vasc Biol* 2009 June;29(6):963-8.

(9) Sanada S, Asanuma H, Minamino T et al. Optimal windows of statin use for immediate infarct limitation: 5'-nucleotidase as another downstream molecule of phosphatidylinositol 3-kinase. *Circulation* 2004 October 12;110(15):2143-9.

(10) Suzuki K, Miura T, Miki T, Tsuchida A, Shimamoto K. Infarct-size limitation by preconditioning is enhanced by dipyridamole administered before but not after preconditioning: evidence for the role of interstitial adenosine level during preconditioning as a primary determinant of cardioprotection. *J Cardiovasc Pharmacol* 1998 January;31(1):1-9.

(11) van Giezen JJ, Sidaway J, Glaves P, Kirk I, Bjorkman JA. Ticagrelor Inhibits Adenosine Uptake In Vitro and Enhances Adenosine-Mediated Hyperemia Responses in a Canine Model. *J Cardiovasc Pharmacol Ther* 2011 June 22.

(12) Ohman J, Kudira R, Albinsson S, Olde B, Erlinge D. Ticagrelor induces adenosine triphosphate release from human red blood cells. *Biochem Biophys Res Commun* 2012 February 24;418(4):754-8.

(13) Riksen NP, Rongen GA, Boers GHJ, Blom HJ, van den Broek PHH, Smits P. Enhanced Cellular Adenosine Uptake Limits Adenosine Receptor Stimulation in Patients With Hyperhomocysteinemia. *Arterioscler Thromb Vasc Biol* 2005 January 1;25(1):109-14.

(14) Riksen NP, Oyen WJ, Ramakers BP et al. Oral therapy with dipyridamole limits ischemia-reperfusion injury in humans. *Clin Pharmacol Ther* 2005 July;78(1):52-9.

(15) Teng R, Mitchell PD, Butler KA. Pharmacokinetic interaction studies of co-administration of ticagrelor and atorvastatin or simvastatin in healthy volunteers. *Eur J Clin Pharmacol* 2012 August 25.

(16) Butler K, Teng R. Evaluation and characterization of the effects of ticagrelor on serum and urinary uric acid in healthy volunteers. *Clin Pharmacol Ther* 2012 February;91(2):264-71.

(17) Teng R, Butler K. Pharmacokinetics, pharmacodynamics, tolerability and safety of single ascending doses of ticagrelor, a reversibly binding oral P2Y(12) receptor antagonist, in healthy subjects. *Eur J Clin Pharmacol* 2010 May;66(5):487-96.

(18) Riksen NP, Franke B, van den BP, Naber M, Smits P, Rongen GA. The 22G>A polymorphism in the adenosine deaminase gene impairs catalytic function but does not affect reactive hyperaemia in humans in vivo. *Pharmacogenet Genomics* 2008 October;18(10):843-6.
